# Retinotopic organization of extrastriate cortex in the owl monkey—dorsal and lateral areas

**DOI:** 10.1017/S0952523815000206

**Published:** 2015-09-14

**Authors:** MARTIN I. SERENO, COLIN T. MCDONALD, JOHN M. ALLMAN

**Affiliations:** 1 Division of Biology 216-76, California Institute of Technology, Pasadena, California 92115; 2 Cognitive Science, University of California, San Diego, La Jolla, California 92093-0515; 3 Experimental Psychology, University College London, London WC1H 0AP, UK.; 4 Department of Psychological Sciences, Birkbeck College University of London, London WC1E 7HX, UK.

**Keywords:** Retinotopy, Polar angle, Eccentricity, Visual field sign, Owl monkey

## Abstract

Dense retinotopy data sets were obtained by microelectrode visual receptive field mapping in dorsal and lateral visual cortex of anesthetized owl monkeys. The cortex was then physically flatmounted and stained for myelin or cytochrome oxidase. Retinotopic mapping data were digitized, interpolated to a uniform grid, analyzed using the visual field sign technique—which locally distinguishes mirror image from nonmirror image visual field representations—and correlated with the myelin or cytochrome oxidase patterns. The region between V2 (nonmirror) and MT (nonmirror) contains three areas—DLp (mirror), DLi (nonmirror), and DLa/MTc (mirror). DM (mirror) was thin anteroposteriorly, and its reduced upper field bent somewhat anteriorly away from V2. DI (nonmirror) directly adjoined V2 (nonmirror) and contained only an upper field representation that also adjoined upper field DM (mirror). Retinotopy was used to define area VPP (nonmirror), which adjoins DM anteriorly, area FSTd (mirror), which adjoins MT ventrolaterally, and TP (mirror), which adjoins MT and DLa/MTc dorsoanteriorly. There was additional retinotopic and architectonic evidence for five more subdivisions of dorsal and lateral extrastriate cortex—TA (nonmirror), MSTd (mirror), MSTv (nonmirror), FSTv (nonmirror), and PP (mirror). Our data appear quite similar to data from marmosets, though our field sign-based areal subdivisions are slightly different. The region immediately anterior to the superiorly located central lower visual field V2 varied substantially between individuals, but always contained upper fields immediately touching lower visual field V2. This region appears to vary even more between species. Though we provide a summary diagram, given within- and between-species variation, it should be regarded as a guide to parsing complex retinotopy rather than a literal representation of any individual, or as the only way to agglomerate the complex mosaic of partial upper and lower field, mirror- and nonmirror-image patches into areas.

## Introduction

Visual areas dominate the neocortex in primates, occupying more than half of its total area. Auditory, somatosensory, motor, and limbic areas all fit into the remaining half. One major task has been to define the borders of these areas. For areas beyond V1, V2, and MT (see Appendix for abbreviations), this has proved to be a surprisingly difficult task. Several different summary diagrams of visual areas in several different nonhuman primates have been published (e.g., Allman, 1977; Felleman & Van Essen, 1991; Kaas & Krubitzer, 1991; Sereno & Allman, 1991; Distler et al., 1993; Rosa et al., 1993; Rosa et al., 2009; Janssens et al., 2013). There are a number of differences between these maps, even within the same species. Beyond V1, V2, and MT, anatomical and physiological studies have been carried forward in the absence of generally agreed-upon area borders, names, neighbor relations, and numbers.

The small size and variability of cortical areas makes it very difficult to build up a coherent picture from small amounts of mapping or connectional data from different individuals. Therefore, we made a particular effort to collect very large contiguous retinotopic mapping data sets in single animals. We quantitatively interpolated and contoured them, analyzed them with the visual field sign technique (Sereno et al., 1994), and then related them to myeloarchitecture or cytochrome oxidase patterns in physical flatmounts of the same cortex.

Visual areas in the cortex are best defined on the basis of converging criteria (Allman & Kaas, 1971*a*; Van Essen, 1985). These include visuotopic organization, architectonic features, connection patterns, neurophysiological properties, and the effects of lesions. While we very much agree with the notion of using converging evidence, we think it is important to first rigorously divide up the cortex on the basis of each measure on its own terms—e.g., retinotopy, myelination, or connection patterns—*before* attempting to optimally combine these maps into a single summary parcellation. The visual field sign technique described below provides a rigorous, objective way to parse the cortex into different areas using retinotopy (eccentricity plus polar angle) alone so that these borders can be compared to those independently revealed by other techniques, such as myeloarchitecture and connection patterns. This contrasts subtly but significantly with an approach where (1) areal borders are first primarily determined using one technique (e.g., flatmounted architectonics), and (2) these borders are used to illustrate data from another technique (e.g., a small number of retinotopic mapping points or injection sites) that is not capable of being used by itself to generate a set of 2-D areal borders. While this latter approach results in a tidier picture, it is less transparent about how information from different techniques—whose power to locally distinguish cortical areas varies across the cortex—has been combined. The visual field sign technique greatly helped to understand the complex retinotopy in the regions we mapped, revealing systematic structure in two dimensions that was difficult to appreciate by examining single penetration rows, or even quantitative isoeccentricity or isopolar angle maps by themselves.

Preliminary reports on this work have appeared elsewhere (Sereno et al., 1986, 1987; Sereno & Allman, 1991).

## Materials and methods

We recorded single- and multiunit responses to visual stimuli from cortical areas in seven hemispheres of six acutely anesthetized owl monkeys (*Aotus trivirgatus*) of both sexes weighing between 0.9 and 1.2 kg. The present report is confined to the results obtained in five of the seven hemispheres where extensive mapping data were obtained (one sagitally sectioned, four flatmounts). A subsequent report will concentrate on results obtained in a separate set of five chronic anesthetized mapping experiments. We consulted a library of 50 acute mapping experiments on which the original model of cortical visual areas in owl monkeys was based (Allman & Kaas, 1971*a*, 1971*b*, 1974, 1975, 1976) to guide our experiments.

### Acute mapping experiments

Our acute anesthetized recording techniques are described in detail in Sereno et al. (1994) and were approved by the Caltech animal experimentation ethics committee. Briefly, under deep anesthesia (initial 40 mg/kg ketamine I.M. and 6 mg/kg I.M. triflupromazine, ketamine supplemented as needed), we retracted the scalp, attached a support post to the skull with dental acrylic and stainless steel screws, and made a craniotomy over extrastriate cortex. The dura was retracted and the exposed cortex covered with heavy sterile silicone oil. The angle of gaze was fixed with an eye ring (9.5 mm inside dia., 1.2 mm width bearing surface) machined to match the contour of the cornea that was attached with Histoacryl cyanoacrylate tissue cement to the locally anesthetized corneal margin (0.7% dibucaine in contact lens wetting solution), which combined with the posterior attachment of eye muscles on the large orbit provided excellent gaze stability. The eye was focused on a 28.5 cm diameter translucent plastic hemisphere by retinoscopy using a gas-permeable contact lens. The animal was kept hydrated with intravenous 5% dextrose in saline, its body temperature maintained with a warm water pad, and urine accumulated in the bladder expressed. Pupil dilation was maintained with Cyclogyl (1%) and general anesthesia of the unparalyzed animal was maintained with additional infusion of ketamine (3–5 mg/kg/h I.M., or as needed to suppress muscular and heart rate responses to mildly noxious stimuli) combined with triflupromazine (2 mg/kg I.M. given at 10–12 h intervals because of its longer resident time) to potentiate the effects of ketamine.

Glass-coated platinum-iridium electrodes were driven perpendicularly into the cortex to depths of approximately 700 microns using a stepping motor microdrive (Herb Adams design) positioned in the *x*–*y* plane using a Narishige micromanipulator. Deeper tangential penetrations were made along the medial wall and along the banks of the superior temporal sulcus where new receptive fields were recorded every 200 microns. Small marker lesions (10 mA direct current for 10 s) were made at chosen superficial sites and in every deep penetration. Penetration sites were located and marked on a 20× cortex photograph using blood vessel landmarks. Receptive field boundaries were plotted on the translucent hemisphere while listening to an audio monitor using light and dark spots and bars as stimuli. Fixation of gaze was repeatedly verified by back-projecting the optic disk as well as four retinal blood vessel landmarks onto the hemisphere after every 15–40 receptive fields had been recorded. In all the experiments reported here, these landmarks remained fixed to within our estimated back-projection measurement error (∼1 deg) for the duration of the experiment (50–100 h). Receptive fields and retinal landmarks were periodically copied to hemispherical paper sheets to clear the plastic hemisphere. Penetrations were made in long anterior–posterior rows, at a density of at least 4 penetrations per sq mm (500 micron interpenetration distance).

### Histology

At the end of each experiment, the animal was euthanized with a lethal dose of Nembutal and perfused through the heart with buffered saline. In two hemispheres, this was followed immediately with standard fixative (4% paraformaldehyde, 0.1 m phosphate buffer). In 5 other hemispheres, we first quickly removed the unfixed brain and gently dissected the white matter away from the gray matter with dry cotton swabs (Sereno et al., 1994). The flat cortex was then placed in either 4% paraformaldehyde or 2% glutaraldehyde and held for a short time under weights between glass slides before being allowed to float freely.

Intact and flatmounted cortices were infiltrated with 30% sucrose and sectioned at 50 microns on a large freezing stage and then stained for myelin (Gallyas, 1979), cytochrome oxidase (Tootell et al., 1985), or cell bodies (Cresyl violet). Intact hemisphere sections were alternately stained for myelin and cell bodies. Flatmounted hemispheres were sectioned parallel to cortical laminae, and every section was stained either for myelin (paraformaldehyde fixation) or cytochrome oxidase (glutaraldehyde fixation).

### Myeloarchitecture and cytochrome oxidase plots

Physically flatmounted sections cut approximately parallel to cortical laminae reveal subtle tangential myeloarchitectonic and cytochrome oxidase features in two dimensions. Such features are often visible in one dimension in conventional sections but are difficult to reconstruct in their entirety in two dimensions from (a much larger number of) conventional sections, especially if these features are small or have undulatory borders. Accurately determining areal boundaries can be difficult when a conventional section passes through the edge of the area, even in “easy” cases like MT (e.g., the lateral border of MT in sagittal sections). On the other hand, single sections from flatmounts can pass through more than one cortical layer in different parts of the section. Because of this, we made 10× pencil drawings of the overall density of the Gallyas myelin stain and the cytochrome oxidase stain that superimposed all the sections from one hemisphere using a microfilm reader (Aus Jena) to project the images and radial blood vessels to align the sections. These stacked drawings were scanned and then used directly as a background for illustrations, or as templates for schematic diagrams of myelin or cytochrome oxidase density.

### Digitization and analysis of recording sites and receptive fields

**Fig. 1.**
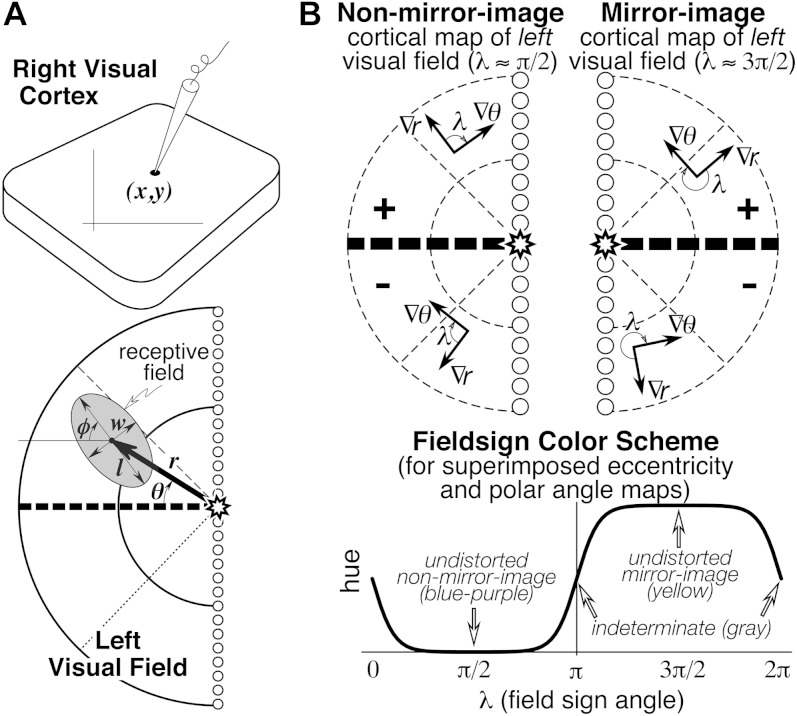
(**A**) Seven receptive field parameters (digitized on a sphere). Receptive field center is defined by (*r*, *θ*), size, and shape by (*l*, *w*, *φ*), and cortical recording site location by (*x*, *y*). An arrow diagram is made by placing a scaled copy of the thick arrow from the center of gaze (star) to the receptive field center at the *x*–*y* position on the cortex where that receptive field was recorded. (**B**) Local visual field sign of a cortical retinotopic map of the *left* hemifield is the (clockwise) angle, *λ*, between the direction of the eccentricity gradient (grad *r*) on the cortex, and the direction of the polar angle gradient (grad *θ*) (lower to horizontal to upper) on the cortex. An angle near 90 deg (0 < *λ* < *π*) signifies a nonmirror image map of the left hemifield while an angle near 270 deg (*π* < *λ* < 2*π*) signifies a mirror image map of the left hemifield. Visual field sign is invariant to rotations and distortions of cortical maps but also invariant to receptive field coordinate transformations; only relative receptive field positions must be known to compute it. A sigmoidal shading scheme that marks relatively undistorted mirror image regions yellow, relatively undistorted nonmirror image regions blue–purple, and regions of indeterminate visual field sign (near 0 or *π*) gray is shown at the bottom right.

Seven numbers were recorded to digitize each receptive field: the location of the recording site on the cortex (*x*, *y*), the eccentricity (*r*) and angle (*θ*) of the receptive field center relative to the center of gaze, and the length (*l*), width (*w*), and angle (*φ*) of the receptive field ellipse (see Fig. 1A). The first two numbers were obtained from the marked cortex photograph. The latter five numbers were obtained after placing the hemispherical paper receptive field sheets back onto the back-illuminated plastic hemisphere, which had a spherical polar coordinate system (with its “North Pole” at the center of gaze) drawn on it. Sheets were aligned with each other using the back-projected retinal landmarks recorded on each. The position of the horizontal and vertical meridians was estimated using the blind spot and the pattern of vertical and horizontal meridian reversals (see Discussion).

These data sets were analyzed using techniques introduced by Sereno et al. (1994). Command line utilities converted the ASCII table receptive field data files into seven kinds of PostScript files: receptive field charts, numbered penetration charts, arrow diagrams, diameter diagrams, interpolated isoeccentricity and isopolar angle maps, and visual field sign maps. C source code for these utilities and a shell script to run them and GMT (see next) are available at http://www.cogsci.ucsd.edu/∼sereno/.tmp/dist/rfutils/. The isoeccentricity, isopolar angle, and visual field sign maps were contoured and shaded using GMT 3.0 (Wessel & Smith, 1991, 1993), a free software package (for Unix systems) for generating a number of different kinds of PostScript output maps from ASCII table inputs (http://gmt.soest.hawaii.edu/, v3.0 available at ftp://ftp.soest.hawaii.edu/gmt/legacy). The six kinds of PostScript output files were opened with Mac OS X Preview to convert them to PDF and were then assembled, layered, masked, and annotated in Adobe Illustrator.

For flatmounted cases, a deformable template algorithm (in rfutils) was first used to stretch the *x*–*y* locations taken from the photographed penetration map according to the final stained-section location of lesions (see Sereno et al., 1994). Deep penetrations, made visible in the flatmounts by turning on lesion current during electrode withdrawal, were then appended.

### Receptive field charts

The flat hemifield chart that we use represents radial distances (from the center of gaze) faithfully but expands distances in a circumferential direction more and more as one moves away from the center of gaze; the circumferential stretching ranges from no distortion at the center of gaze up to a circumferential magnification of *π*/2 (∼1.57×) at 90 deg eccentricity. Each receptive field was therefore flat-corrected on the planar visual field map by stretching it in a circumferential direction as a function of its eccentricity to accurately represent receptive field overlap and position of receptive field borders relative to isoeccentricity and isopolar angle landmarks on the flat hemifield chart.

As receptive field size at a particular eccentricity increases, there is an increasing tendency for receptive field reversals to occur when the edge of a receptive field—as opposed to its center—reaches the vertical or horizontal meridian. We did not correct for this. Since the contour maps represent the coordinates of receptive field centers, reversals in higher visual areas can occur at some distance from the vertical or horizontal meridian even though receptive fields in that area cover the entire quadrant. We implemented a conservative receptive-field-size dependent meridian correction method (available in software package described above) but found that it had virtually no effect on the position of visual field sign borders, even though polar angle map reversals more closely approached meridians (results not shown).

For ease of interpretation, all cortical hemispheres are represented as right hemispheres and corresponding receptive fields are all illustrated in the left hemifield. The angles of the left hemifield receptive field centers are measured in a clockwise direction starting from the left horizontal meridian (the angle of the receptive field ellipse is treated similarly) so a receptive field in the upper left visual quadrant will have an angle between 0 and 90 deg, while a receptive field in the lower left visual quadrant will have an angle between 0 and −90 deg.

### Arrow diagrams

The complete raw receptive field data for each case can be displayed in the form of an arrows diagram. The visual field location of each *receptive field center* is represented as a scaled arrow whose center is placed at the *x*–*y* position on the cortical surface from which it was recorded (see bold arrow at bottom of Figs. 1A and 5). The angle and length of each arrow represents the angle and distance of the *receptive field center* from the center of gaze (not direction selectivity). Thus, a peripheral receptive field on the horizontal meridian would be represented as a long horizontal arrow while a receptive field on the upper field vertical meridian near the center of gaze would be a short upward-pointing arrow.

### Interpolated isoeccentricity and isopolar angle maps

The eccentricity and angle data were interpolated onto regular grids using a distance weighted smoothing technique (Lancaster & Salkauskas, 1986). The interpolated value ζ_*j*_ at the *j*^th^ *grid point* is the distance–weighted sum of the values, *z*_*i*_, of all the surrounding *i* data points, scaled by the sum of the weights:
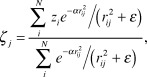
where the weight for the *i*^th^ data point, 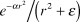, is a Gaussian function of the distance *r* (in mm) between the *j*^th^ grid point and the *i*^th^ data point. The value of *α* controls the width of the Gaussian (larger *α* makes a narrower Gaussian, which more strongly emphasizes nearby points) while *ε* adjusts the height of the Gaussian (larger *ε* makes a less tall Gaussian, which makes the surface stiffer). We used *α* = 0.6–1.2 and *ε* = 0.1–0.2. The resulting interpolated surface followed the data quite closely, trimming only a few degrees from the peaks in *r* and *θ*. Despite its locally smooth appearance, this interpolation scheme smooths over maxima and minima less than the one used in Maunsell and Van Essen (1987) and Neuenschwander et al. (1994) (see Sereno et al., 1994).

### Visual field sign maps

Retinotopic cortical maps consisting of two superimposed contour maps are very hard to read when they contain multiple, distorted visual field representations. However, for each small portion of such a map, one can calculate the sign of the visual field representation—that is, whether it is a nonmirror image or mirror image representation of the retina (when viewed from the cortical surface) (Sereno et al., 1994, 1995). For right hemisphere (left hemifield) data, the visual field sign can be determined from the (clockwise) angle, *λ*, between the direction of the gradient in eccentricity, *r*, and the direction of the gradient in polar angle, *θ* (see Fig. 1B, top), both gradients measured with respect to distance along the cortex. The cortical gradient in eccentricity points in the local (cortical) direction of fastest increase in eccentricity; the polar angle gradient points in the local direction of the fastest increase in polar angle (from lower field to horizontal meridian to upper field). The gradient direction is locally perpendicular to contour lines for that coordinate, pointing uphill. The angle between the gradients, *λ*, is a relative measure unaffected by linear transformations (e.g., rotations, scaling, translations) of the coordinate systems used to digitize both the cortical sites as well as the receptive fields (e.g., *λ*, is unaffected by the placement of the meridians, as long as relative positions of receptive fields are maintained). An angle between the gradient directions of *π*/2 signifies an undistorted (conformal) nonmirror image representation of the left hemifield while angle of 3*π*/2 signifies an undistorted mirror image representation; other angles estimate the amount of local angular distortion in the visual field representation. A binary map of right cortical hemisphere visual field sign can be produced by distinguishing angles of 0 to *π* from angles of *π* to 2*π*. Here we use a smoother, sigmoidal shading scheme—mirror image field sign is yellow, nonmirror image field sign is blue–purple, and regions of indeterminate field sign (*λ* near 0 or *π*) are gray (Fig. 1B, bottom). The gradients in the eccentricity and polar angle maps were estimated over finite differences in the *x* and *y* directions of 500–600 microns to reduce noise.

To improve the readability of the superimposed isoeccentricity and isopolar angle maps, we systematically varied the textures of the different contour lines. To distinguish isoeccentricity from isopolar angle contours, we made the isoeccentricity contours thicker and continuous (there is an extra-thick isoeccentricity contour every 10 deg), while we made the isopolar angle contours thinner, and then dashed or dotted (there is a slightly thicker isopolar angle contour every 20 deg). Dashed isopolar angle contours indicate upper fields (more prominent) while dotted contours indicate lower fields (less prominent). These conventions are respected in every contour plot.

## Results

We illustrate five cases in detail. Data from two additional hemispheres were similar to those reported here but much less complete. The small size of extrastriate visual areas coupled with interanimal variability makes it quite difficult to build up a picture of extrastriate organization by combining small amounts of data from a number of different animals. Extensive data sets in individual animals are physically exhausting to obtain, however, and often span many areas. We were not able to sample all the areas we recorded from here at the same density. Nevertheless, we feel that the advantages of having contiguous data sets from single animals make it worth considering each extensive case in its entirety instead of breaking the data down by areas and presenting them in separate papers. This also reduces the temptation to create “wastebasket” areas.

To aid comparisons, areal labels have been placed in exactly corresponding positions in successive illustrations (e.g., eccentricity, polar angle) of the same data set. Flatmounts reduce a 3D surface to a 2D surface, making it easier to see all areas at once. To ease interpretation, we have oriented all the flatmounts so that they correspond to a lateral view of a conventionally oriented hemisphere. But flatmounts make it somewhat awkward to directly apply positional terms used to describe directions in the 3D brain. Therefore, we will consistently use “medial” to refer to movements that bring a point nearer the posterior cingulate region [this could be superior (dorsal) on the lateral 3D surface or inferior (ventral) on the dorsomedial 3D surface] and “lateral” to refer to movements that bring a point closer to medial temporal lobe structures (usually inferior on the lateral 3D surface).

Three large cases in which we stained the cortex for myelin are first presented in lateral to medial order. Two additional cases in which the cortex was stained for cytochrome oxidase are then presented. Every responsive penetration (almost 1800 in the five cases illustrated here) was included in the isoeccentricity, isopolar angle, and visual field sign maps. To save space, we have only illustrated a fraction of the receptive fields on arrow diagrams and conventional receptive field plots.

### Summary diagram

**Fig. 2.**
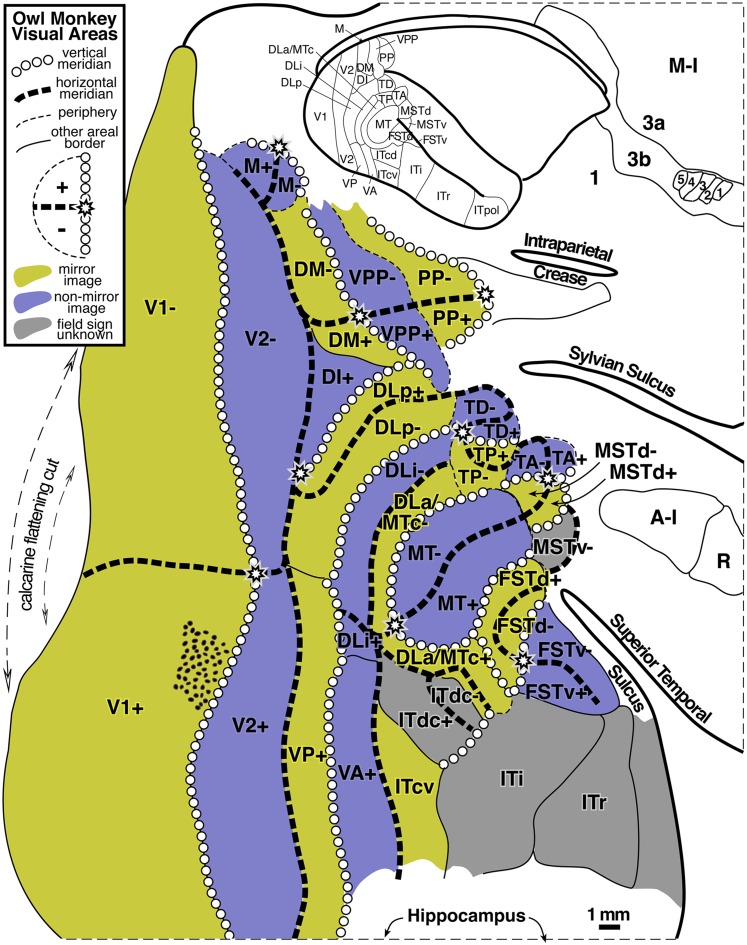
Schematic proposal for the retinotopic organization of 24 owl monkey visual cortical areas drawn using a myelin-stained flatmount. Anterior to V2 near the dorsal convexity of the brain are 3 areas with alternating field sign—DM, VPP, and PP. DM and VPP share a center of gaze and vertical meridian representation. VPP and PP share a periphery representation. The upper fields of DM and VPP curve anteriorly, away from V2. Just lateral to DM, there is another series of strip like areas with alternating field sign—DI (which unlike DM, has the same field sign as V2), DLp, DLi, DLa/MTc, and finally MT. DLa/MTc bends laterally away from MT along a shared vertical meridian border with FSTd. The complex topography anterior to MT is best visualized as two pairs of areas—TD/TP and TA/MSTd. Each pair of areas shares a center of gaze and a vertical meridian representation. In all four areas, the upper field is anterior to the lower field. Anterior and lateral to MT are FSTd and FSTv, which share a center of gaze and a vertical meridian. ITcd contains mainly an upper field representation lateral to DLa/MTc. Retinotopy of M, ventral VP and VA, and ITi, and ITr were taken from Allman and Kaas (1975), Newsome and Allman (1980), and Weller and Kaas (1987).

**Fig. 3.**
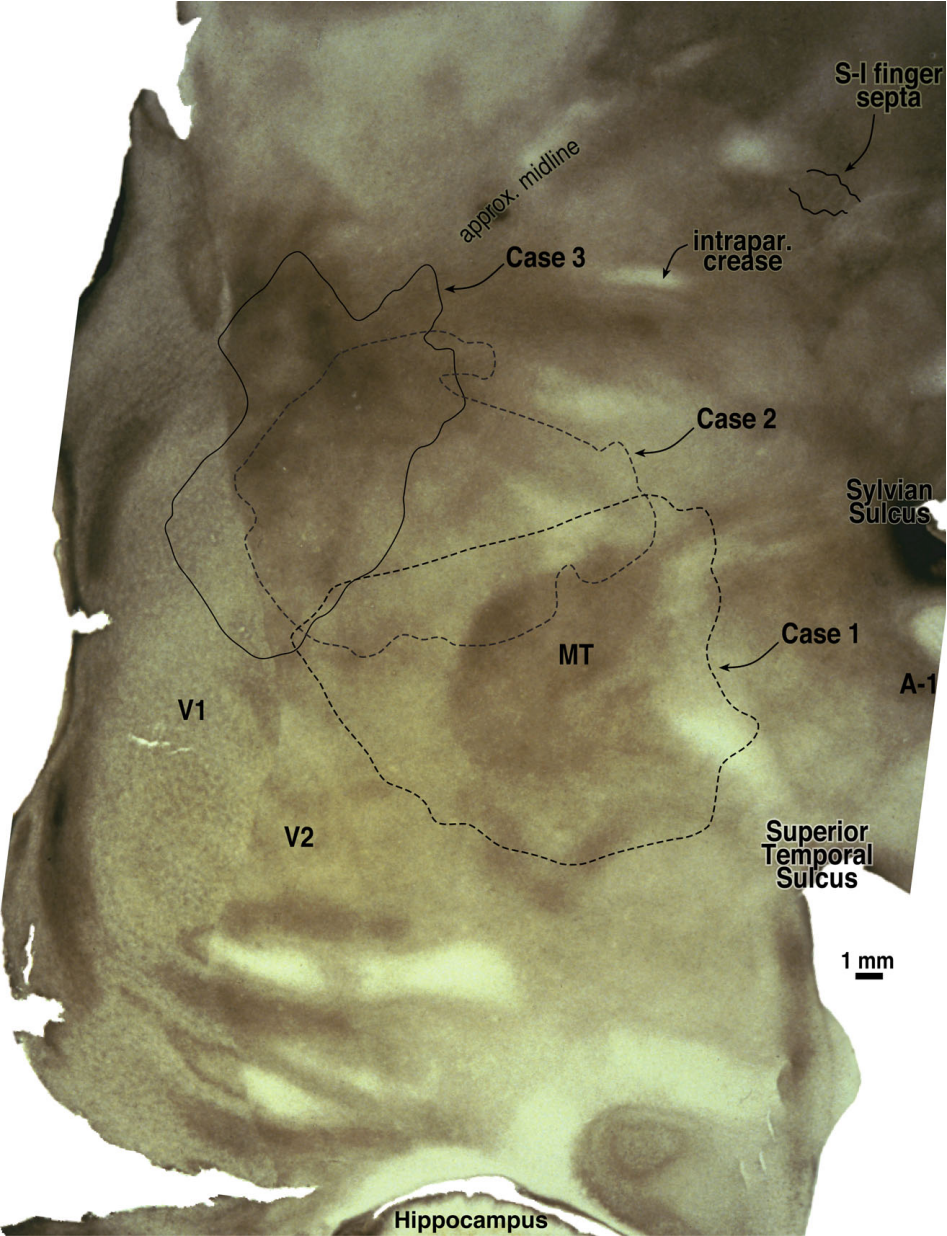
Low power view of case 3 flatmounted cortex (see Fig. 13 for close-up). The exact location of case 3 recording area (solid line) and the approximate locations of cases 1 and 2 recording areas are illustrated (dashed lines) with less obscuring annotation than in Fig. 13. Most of right cortical hemisphere is visible here, except for the frontal and temporal poles. Two slight ripples in this flatmount in posterior inferotemporal cortex caused uneven sampling of cortical laminae there (1–2 mm width horizontal light and dark artifacts below “V2” label). This flatmount was used as the basis for the summary diagram.

A summary of the retinotopic organization of owl monkey extrastriate cortex is shown in Fig. 2. It was drawn using the myelin-stained flatmount from case 3 (see Fig. 3). The conventions used for the meridians—vertical meridian marked by circles, and horizontal meridian marked by thick dashes—follow earlier studies (e.g., Allman & Kaas, 1971*a*; Allman & Kaas, 1975; Newsome et al., 1986; Desimone & Ungerleider, 1986; Gattass et al., 1988; Boussaoud et al., 1991; Sereno et al., 2012; note that Rosa et al., 1988, 1993; 2005; 2009, Fiorani et al., 1989, and Neuenschwander et al., 1994 have reversed this convention, while Janssens et al., 2013 and Kolster et al., 2014 use yet a different one). In addition, the center of gaze is marked by a star and the periphery by thin dashes. These notations have been used rather loosely, as is the custom in the literature, to indicate the orientation (essentially the visual field sign) of a retinotopic patch rather than as substitutes for the actual contours, which are illustrated in the following figures at great length. In a number of cases, the re-representation of the visual field in an area is incomplete and distorted.

The retinotopic organization of owl monkey dorsal and lateral extrastriate cortex beyond V2 uncovered in the present experiments can be schematized as medial, intermediate, and lateral clusters of strip-like areas with alternating field sign, then area MT, and then a series of small areas anterior to MT. At the dorsal convexity of the brain is the first cluster of strip-like areas consisting of DM (mirror), VPP (nonmirror), and PP (mirror). Perhaps DI (nonmirror, same as V2) also belongs to this cluster. Immediately lateral to the medial cluster is a second cluster consisting of DLp (mirror), DLi (nonmirror), and DLa/MTc (mirror). The third, lateralmost cluster includes VP (mirror), VA (nonmirror), and possibly ITcv (mirror). MT (nonmirror) is in the center of extrastriate cortex. The small areas beyond MT are easiest to understand in pairs—first TD/TP and then TA/MSTd. Those pairs each share a center of gaze and a vertical meridian representation. In all four areas, the upper field is approximately anterior to the lower field. Anterior and lateral to MT are a third pair of areas—FSTd and FSTv, which share a center of gaze and a vertical meridian. Parietal and temporal areas not yet mapped in detail lie anterior to the first and third series of areas.

Fig. 3 shows a myelin-stained flatmount sections from case 3 showing the location of the recordings in case 3, along with the approximate locations of the recording areas in cases 1 and 2 (see below for detailed description).

### Case 1—lateral cortex (370 sites)

**Fig. 4.**
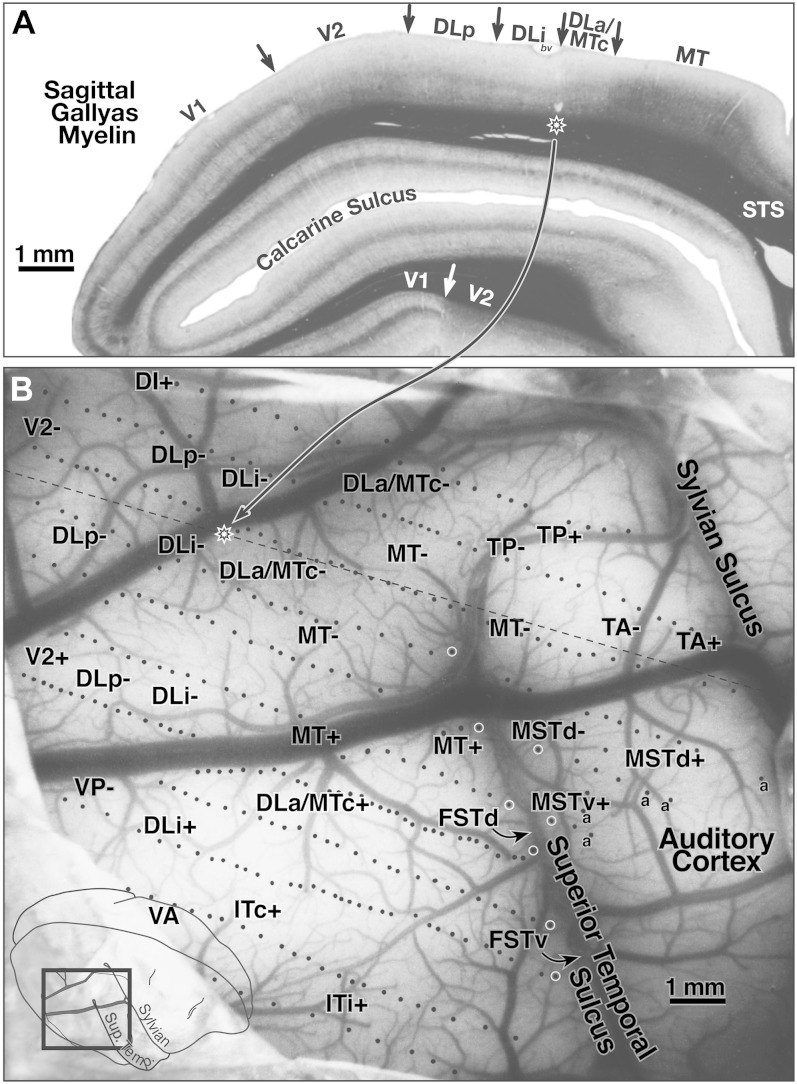
(**A**) Myelin-stained sagittal section from case 1 with electrophysiological borders. V1 is identified by the dense band of myelin in layer 4B. V2 is relatively densely myelinated in this section. Myelination is reduced on entering DLp. There is a further subtle reduction in myelination and thinning of intermediate and lower cortical layers upon entering DLi. The electrophysiological border of DLi with DLa/MTc (2 lesions above star) as myelin intensifies entering DLa/MTc. There was a sharp increase in myelination and a further thickening of the intermediate and lower layers entering MT. Myelination drops in peripheral MT just after it bends down into the superior temporal sulcus. (**B**) Surface location of visually responsive recording sites for case 1 on a photo of exposed cortex at same magnification as sagittal section in **A**. Position of magnified view is shown in lower left inset. White circles along the superior temporal sulcus mark deeper penetrations containing multiple recording sites. The nearly horizontal black dashed line indicates the position of the myelin-stained sagittal section (star on dashed line shows position of star in sagittal section). Auditory-only responses are marked “a”. The superior temporal sulcus was unfolded in subsequent illustrations to bring deep recording sites to the surface. Locations of area labels are preserved in succeeding maps to aid comparisons.

**Fig. 5.**
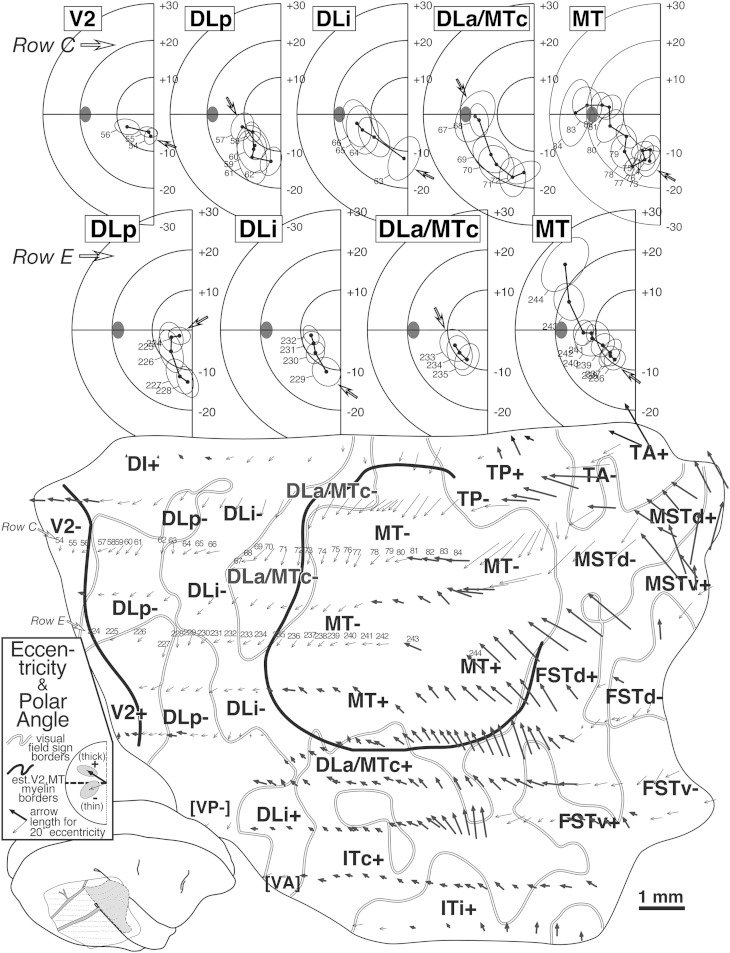
Arrow diagram (bottom) for case 1 summarizing retinotopy with selected receptive field rows (top). Each of 350+ visually responsive recording sites is marked by an arrow whose *length* indicates the eccentricity of the receptive field center and whose *angle* indicates the polar angle of the receptive field center for that site (see legend for arrow scale). Upper field arrows are bold to emphasize upper *versus* lower. The unfolding of the superior temporal sulcus has caused the rows of superior temporal gyrus points to curve upwards anterior to MT. Thick black lines are myeloarchitectonic borders of V2 and MT, while medium gray lines are visual field sign transitions traced from Fig. 8. At the top, two penetration rows (numbered arrows in row C and E) are illustrated as conventional receptive field plots. Small open arrows in each plot indicate receptive field corresponding to the most posterior penetration in each area (filled ellipse: optic disk). Row C (sites 54–85) sampled 5 areas. Beginning in V2, receptive fields approach the horizontal meridian, reverse on entering DLp, reverse again at the vertical meridian entering DLi, again at the horizontal meridian on entering DLa/MTc, and again at the vertical meridian entering MT. Row E (sites 224–244) sampled four areas, beginning with DLp. A parallel series of reversals is seen, except that receptive fields are more central than their fellows from the previous row.

**Fig. 6.**
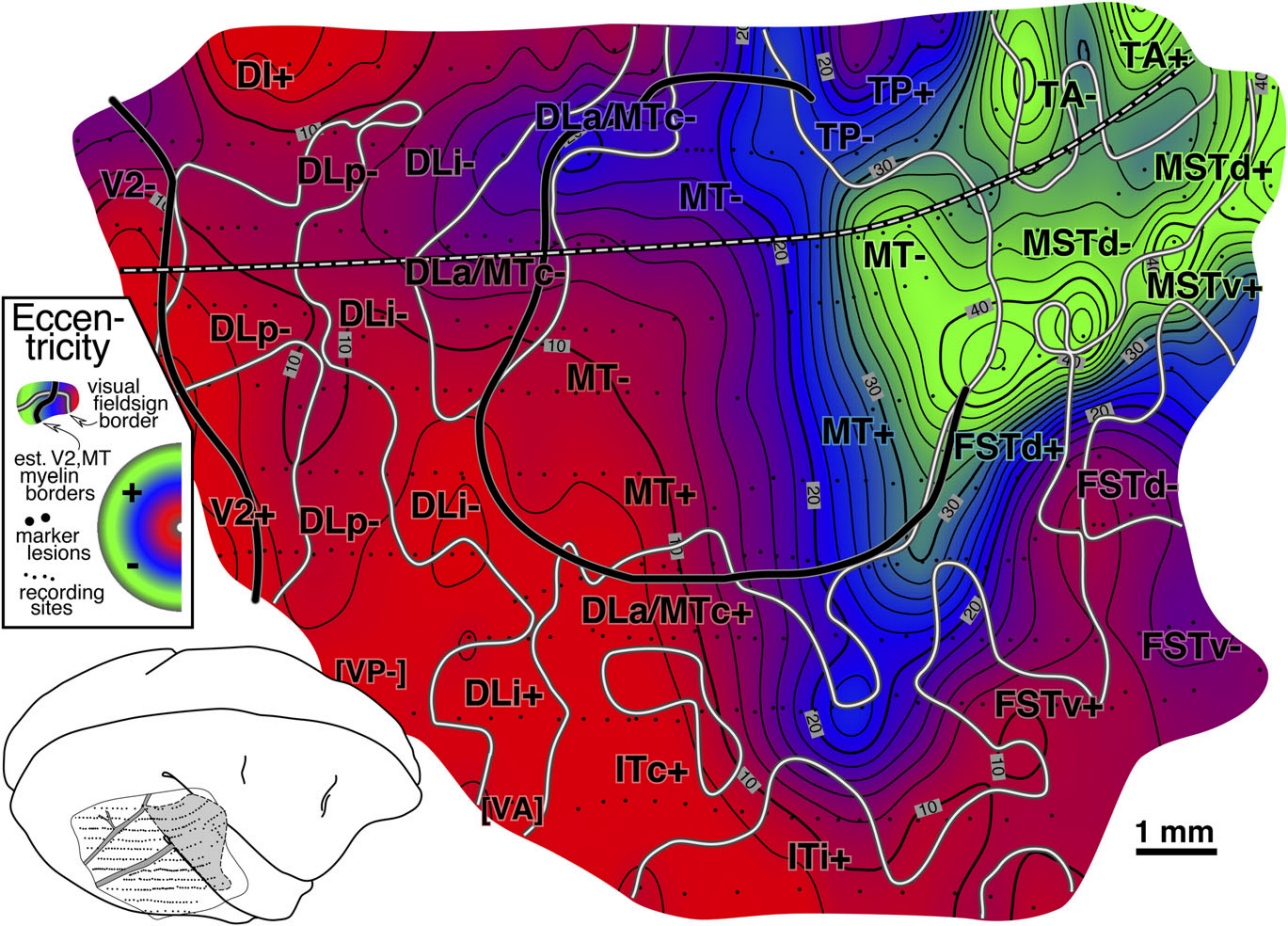
Cortical map of receptive field *eccentricity* for case 1. Receptive field eccentricity was interpolated onto a regular grid and contoured (interval: 2 deg). The contour map was shaded (central eccentricities are red, middle eccentricities blue, and peripheral ones green). There are several eccentricity minima (e.g., center of gaze of V2, DI, MT, and FSTd) and eccentricity maxima (e.g., MT periphery, TP/TA border). Almost parallel isoeccentricity lines in MT indicate a particularly orderly visual field representation there. The course of the sagittal section from Fig. 4A is indicated by the black-rimmed dashed line.

**Fig. 7.**
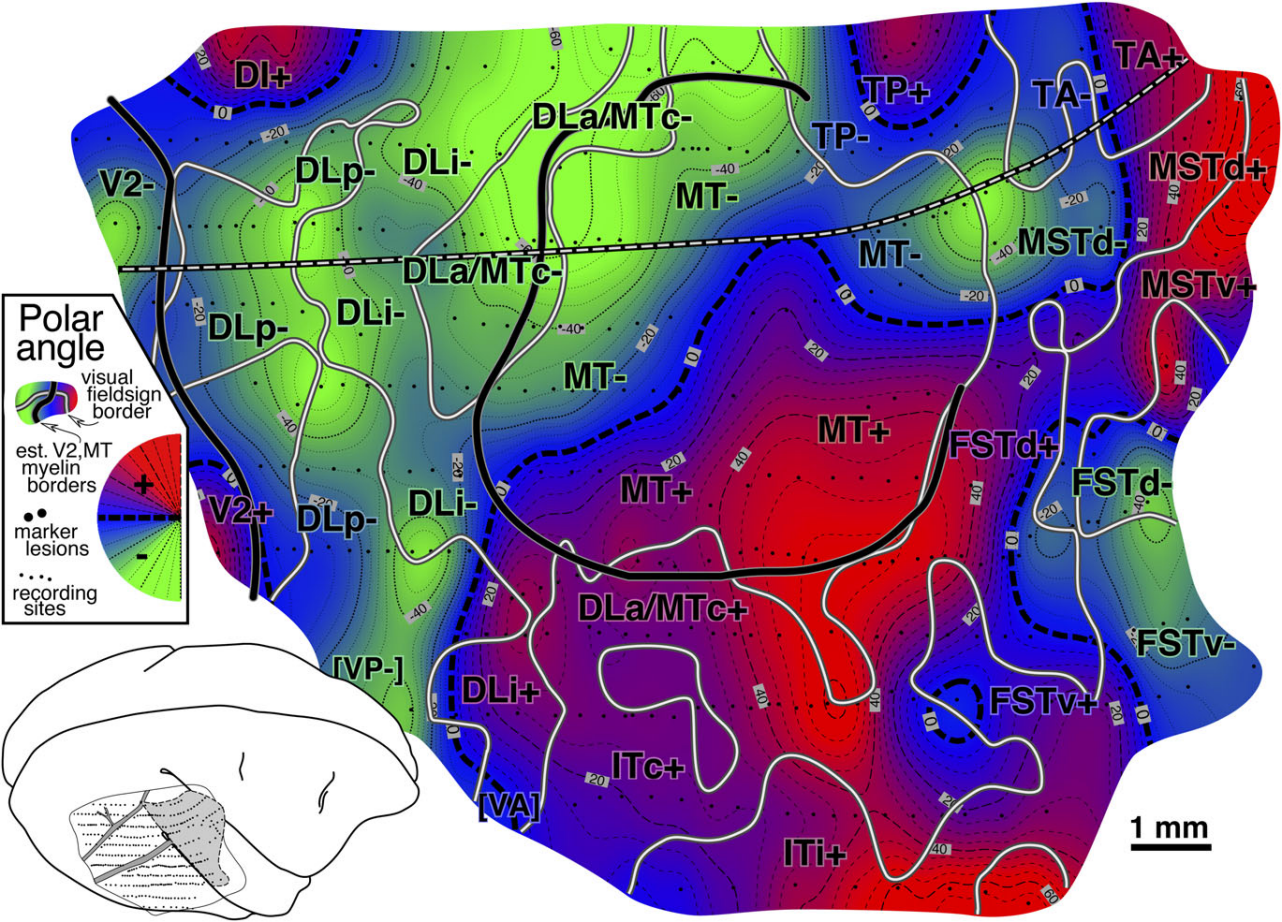
Cortical map of receptive field *polar angle* for case 1. Receptive field polar angle was interpolated and contoured (interval: 5 deg) using the same parameters as in the previous Figure, and then shaded (lower field is green, horizontal meridian blue, and upper field is red). Lower field contours use thin *dotted* lines, upper field contours use thin *dashed* lines, and the horizontal meridian is a thick dashed line. There are several lower field vertical meridians (e.g., posterior border of V2, the DLp/DLi border, and the medial border between DLa/MTc and MT) and several upper field meridians (e.g., the lateral border of MT; this meridian is T-shaped with the bottom of the “T” extending laterally out from MT). There are several horizontal meridians (e.g., the V2/DLp border, the DLi/DLa border, the MT horizontal meridian). Note that the horizontal meridian zero contour (exact horizontal meridian) will not generally appear at a typical horizontal meridian reversal where receptive field return to the same quadrant; it will surely appear only if receptive fields cross into the opposite quadrant.

**Fig. 8.**
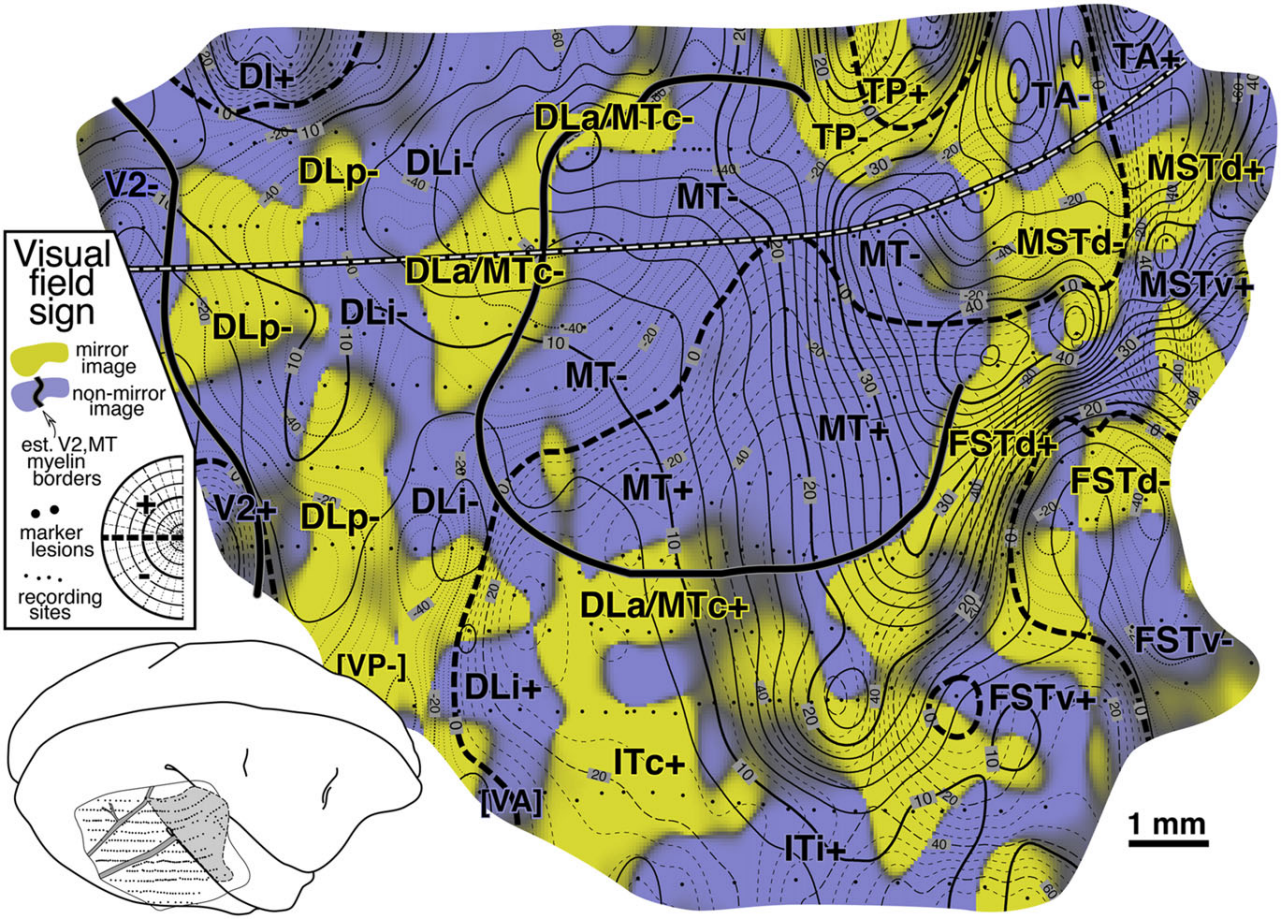
Cortical map of *visual field sign* for case 1. Nonmirror image cortex is shaded blue–purple and mirror image cortex is shaded yellow. Contour maps of eccentricity and polar angle from Figs. 6 and 7 used to calculate field sign are both included (isoeccentricity contours are thicker than isopolar angle contours). MT stands out as a large island of nonmirror image cortex with almost orthogonal isoeccentricity and isopolar angle contours, which indicates it is approximately a conformal (angle preserving) map of the visual field. An anteroposterior traverse from “V2-” to “FSTd-” reveals 6 patches of cortex with alternating field sign—V2 (nonmirror), DLp (mirror), DLi (nonmirror), DLa/MTc (mirror), MT (nonmirror), FSTd (mirror). Field sign defined additional areas in parietal cortex (TP, TA, MSTd) and temporal cortex (ITc, ITi) (see text).

Fig. 4B shows the surface location of the recording sites for case 1 on a photo of the exposed cortex (taken through the protective silicone oil bath). The position of the craniotomy is shown in the inset at the lower left. White circles along the superior temporal sulcus indicate the location of deeper penetrations containing multiple recording sites. The dashed line in Fig. 4B indicates the approximate position of the same-magnification myelin-stained sagittal section shown in Fig. 4A. The star on the dashed line indicates the location of a pair of lesions visible in the section. This case covered all the area between lateral V2 and MT and extended anteriorly to the sharp border of visual cortex with auditory cortex on the posterior superior temporal gyrus. Auditory-only responses are marked with an “a” in Fig. 4B. The transition from visual-only to auditory-only responses was surprisingly abrupt; the region of cortex responding well to both visual and auditory stimuli was less than 500 microns wide. The superior temporal sulcus was opened in subsequent illustrations to bring the deep recording sites to the surface, resulting in a gradual upward curving of the anterior–posterior recording site rows on the superior temporal gyrus (Figs. 5–8).

#### Myeloarchitecture (Fig. 4A)

**Fig. 9.**
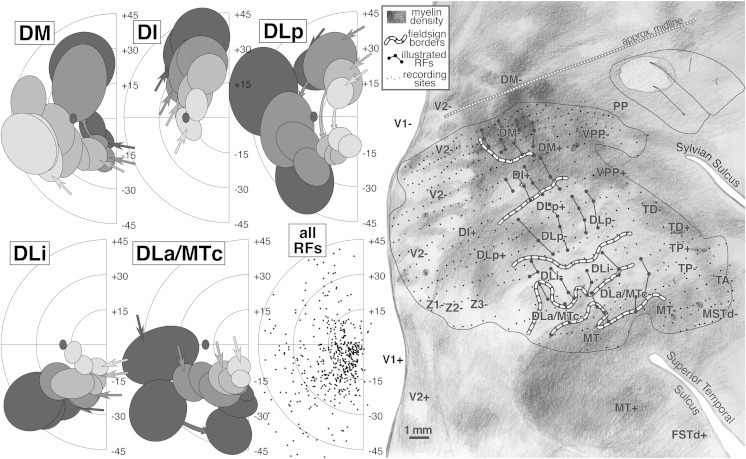
Location of all recording sites for case 2 superimposed on a drawing of myelin-staining in flatmounted sections (right), and receptive field plots for selected receptive field rows (left). The mapped region was medial and posterior that in case 1 (MT only partially exposed, more V2 coverage). The myelin drawing was collapsed across all cortical layers by aligning sections using radial blood vessels. A selected set of field sign borders are indicated with back-rimmed dashed lines. Four rows of recording sites from each of five areas (DM, DI, DLp, DLi, and DLa/MTc) are marked by black lines; corresponding receptive fields from each area are at the left. Receptive field shading indicates which row: the posterior rows are the lightest and the most anterior rows the darkest. Filled arrows indicate the medial (at top) start if each row. A curious visual field sign reversal in the middle of the upper quadrant is visible in the DM and DI receptive field plots. Receptive fields move into the upper field in DM with the light (posterior row) receptive fields the most eccentric. Partway into the upper field, however, the light receptive fields become the *least* eccentric, in DI. The field sign alternations that define the remaining three areas are more conventionally generated by reversals approximately at the vertical or horizontal meridian, and the remaining three areas (DLp, DLi, DLa/MTc) all have the lightest row of receptive fields the least eccentric (like DI). In DLp, receptive fields jump across the horizontal meridian without overlapping. This may be a real discontinuity but could also reflect undersampling.

The most prominent areal boundary in the sagittal section in Fig. 4A is V1, which is clearly distinguishable in a myelin stain because of its peculiar middle layers. The posterior boundary of MT is distinct in this section as well, and it corresponds exactly with a electrophysiologically defined receptive field reversal, which is marked by an arrow. There is a sharp drop in the density of myelination within MT as it enters the superior temporal sulcus, however, that does not correspond to an obvious electrophysiological border (originally reported by Allman & Kaas, 1971*a*; Desimone & Ungerleider, 1986). This lighter part of MT is visible in the flatmounted myelin-stained sections (see Figs. 3 and 9). Just posterior to (left of) MT in Fig. 4A is somewhat less densely myelinated strip of cortex labeled area “DLa/MTc”. The posterior border of DLa/MTc was marked by a pair of lesions and corresponds with a reduction in myelination as one moves posteriorly from DLa/MTc into DLi. The electrophysiologically defined border between DLi and DLp corresponds with a subtle increase in myelination upon moving further posteriorly from DLi into DLp. Finally, the electrophysiological DLp/V2 border is visible as a somewhat more striking increase in myelination as one enters V2 in this section. In other sections, however, the electrophysiological DLp/V2 border was extremely subtle. The V2 myelin pattern in flatmounts shows some evidence of stripes (see Figs. 3, 9, and 13), and the less densely myelinated V2 stripes can be difficult to distinguish from DLp in a sagittal section.

#### Arrow diagram, receptive fields (Fig. 5)

The bottom of Fig. 5 contains an arrow diagram for all the responsive sites in case 1. An arrow diagram compactly represents both coordinates of retinotopy (eccentricity and polar angle) making it possible to examine the grain and internal consistency of a large data set at once (the over 350 receptive fields illustrated here would impossibly clog a standard receptive field chart). Two additional kinds of boundaries have been marked on this and the following illustrations of this case. The gray lines represent the transitions between nonmirror and mirror visual field sign (traced from Fig. 8) while the thick black lines represent the estimated position of the anterior V2 border and the MT border as reconstructed from myelin-stained sagittal sections like the one in Fig. 4A. In general, there is a tendency for the lower visual field (thin arrows) to be represented medially and the upper visual field (thick arrows) to be represented laterally. Eccentricity tends to increase (arrows get longer) as one moves anteriorly. The details of the pattern of change in arrow direction and size, however, are quite complex, and can only be fully appreciated after visual field sign has been calculated.

The arrows in two rows of penetrations (C and E) in Fig. 5 have been numbered and the corresponding receptive fields are illustrated at the top of the Figure, where the open arrows now indicate the start (posteriormost penetration) in each area for that row. Four re-representations of the lower visual field are apparent from the receptive field sequences for Row E (224–244). Receptive fields begin near the horizontal meridian in DLp and move toward the vertical meridian. They then turn back toward the horizontal meridian in DLi. They reverse once again to approach the vertical meridian in DLa/MTc. Finally, they return to the horizontal meridian and continue up into the upper visual field in MT. The more medially situated row C (54–83) begins further posteriorly, in V2, where receptive fields pass from near the vertical meridian to the horizontal meridian. This row then shows the same sequence of reversals described for the previous one, as the row passes through DLp, DLi, DLa/MTc, and finally into MT, except that receptive fields in the corresponding areas are generally about 5 deg more eccentric than in the previous row. Clearly, much of the lower visual field is re-represented within each of these areas.

#### Isoeccentricity and isopolar angle maps (Figs. 6 and 7)

An interpolated contour plot of receptive field *eccentricity* (arrow length) for case 1 is shown in Fig. 6. Visual field sign transitions and estimated myeloarchitectonic borders of V2 and MT are marked on the contour map. The contour map has been shaded to make it easier to read, with the center of gaze red, middle eccentricities blue, and the periphery green (color scheme after Sereno et al., 1995). There are three eccentricity minima (brightest red, most central)—the first at the far posterior extent of the craniotomy near the transition from lower to upper fields in V2, the second near the center of gaze representation of MT (directly under the lateralmost “DLi-” label), and the third at the posterior medial corner of the craniotomy in DI+. Eccentricity generally increases as one moves anteriorly. It reaches a maximum before the anterior border of extrastriate cortex is reached, however, and drops off again as one moves medially (in TP), anteromedially (in TA), anteriorly (beyond MT in MST and FST), and laterally (in ITc). The pattern of isoeccentricity contours is simplest in MT, where they are almost parallel to each other. The black and white dashed line (approximately horizontal) shows the location of the myelin-stained sagittal section shown in Fig. 4A. The anterior end of the sagittal section line curves medially on the flattened surface because as a result of unfolding the superior temporal sulcus (see inset).

An interpolated contour plot of receptive field *polar angle* for case 1 is shown in Fig. 7. The polar angle color scheme (again after Sereno et al., 1995) is now green (and dotted contour lines) to represent visual field locations nearer the *lower* vertical meridian, blue to indicate visual field locations near *horizontal meridian*, and red (and thin dashed contour lines) to represent visual fields nearer the *upper* vertical meridian. The exact horizontal meridian is indicated by a thick dashed line.

The pattern of polar angle is more complex than that of eccentricity and is described in two main parts—the V2 to MT/FST region, and then the areas medial and lateral to MT. We have already seen the alternation between the vertical and horizontal meridians in one dimension in the receptive field sequences in Fig. 5; the isopolar angle map shows this pattern in two dimensions. There is a lower field vertical meridian representation (green) at the extreme posterior edge of the craniotomy, which is near the V1 border. There is a second somewhat undulatory lower field vertical meridian representation (green) along the DLp/DLi border that extends from the medial to the lateral edge of the exposure. This is likely the location of the prominent callosal band revealed within greater DL in the study of Newsome and Allman (1980, their Fig. 8) and Cusick et al. (1984, their Figs. 2–7). There is a third lower field vertical meridian representation (green) along the medial DLa/MT border. Finally, there is an upper field vertical meridian representation (red) at the anterolateral border of MT.

Intercalated with the four vertical meridian representations are four representations of receptive fields near the horizontal meridian (blue). Starting posteriorly, receptive fields at the anterior V2 border come very close the horizontal meridian. Note that the thick horizontal meridian zero-contour (and fully saturated blue) will not generally appear at a typical “horizontal meridian” reversal since few receptive field *centers* make it exactly to the horizontal meridian (see e.g., horizontal meridian reversals at the top of Fig. 5); the zero-contour of polar angle will typically *only* appear if receptive fields cross from the lower into the upper visual field or *vice versa*—as they do in MT and FST. Continuing anteriorly, the border between DLi and DLa/MTc is also close to the horizontal meridian. Further anteriorly, there is the horizontal meridian of MT. Finally, there is a fourth horizontal meridian representation beyond MT in FSTd. These eight borders are all roughly parallel to each other. All eight borders were also traversed in the three long posterior-to-anterior penetration rows in case 4 (see Figs. 17 and 18).

Just medial to wide corridor of five lower field representations in V2, DLp, DLi, DLa/MTc, and MT, at the posterior medial corner of the craniotomy, a small patch of *upper* field (red) stands out, in DI, directly adjoining the lower field V2 border. This medial upper field region was exhaustively mapped in cases 2 and 3.

We turn now to the areas anterior to the lower field V2/MT corridor just described. The medial (lower field, green) border of MT can be divided into two parts. As just described, the posterior part is adjoined medially by lower field DLa/MTc; but the anterior part is directly adjoined by an another lower field representation, TP-. Just medial to TP-, receptive fields move rapidly into the upper field in TP+ (they also move toward the center of gaze—see Fig. 6). Continuing anteriorly beyond TP, receptive fields cross the horizontal meridian back into the lower field in TA only to cross once again back into the upper field in TA and MSTd, where they almost reach the upper field vertical meridian at the medial anterior corner of the craniotomy.

Laterally, the MT border is defined by the upper field vertical meridian (red). Some parts of the border (e.g., in between the lateralmost “MT+” and “DLa/MTc+” labels), however, seem to be at some distance from the vertical meridian. This could be an undersampling artifact—if it was, the near-vertical-meridian portions of both MT+ and DLa/MTc+ in this case would *together* have to occupy a strip of cortex less than 800 microns wide. Just lateral to the “DLa/MTc+” label, receptive fields almost reach the horizontal meridian, which forms the lateral border of DLa/MTc. A little anterior to this, two-thirds of the way along the lateral MT border, there is a prominent upper field vertical meridian representation that is actually elongated in a mediolateral direction—that is, orthogonal to the myeloarchitectonic MT border. This corresponds closely with a strip of callosal connections (Newsome & Allman, 1980, emerging from lateral border of MT in their Figs. 3 and 8; see also Cusick et al., 1984, their Figs. 5 and 7); it appears to represent the DLa/FSTd border. Curiously, it appears that the vertical meridian representation and the callosal band essentially go all the way to MT (that is, the vertical meridian forms a “T” junction at the MT border). Moving anterior to this upper field vertical meridian border between DLa/MTc+ and FSTv+, receptive fields return to the horizontal meridian in FSTv, eventually continuing into the lower field at the far anterior and lateral extent of the craniotomy.

#### Visual field sign map (Fig. 8)

The isoeccentricity and isopolar angle maps each present a complex picture. However, without considering both maps at the same time, it is impossible to detect when the visual field is being re-represented from reversals in one coordinate alone. For example, if the isopolar angle map is sampled along a randomly oriented line (e.g., a penetration row), there will often be several “reversals” of polar angle, some of these near the horizontal or vertical meridians. Without considering the isoeccentricity map, however, it is impossible to distinguish cases where the sampling line crossed out of one area into another visual area (a real reversal) from cases where the line simply came near to and then moved away from a meridian/border, staying within the same area (a pseudo reversal). Unfortunately, double contour maps representing both eccentricity and polar angle are virtually impossible to read when they contain distorted, partial re-representations of the visual field. The visual field sign technique improves readability by providing a well-defined quantitative binary “first cut” of the data into distinct areas.

Fig. 8 shows a visual field sign map for case 1 (mirror image yellow, nonmirror image blue–purple). The most obvious feature of this map is the large central expanse of nonmirror image cortex (blue–purple) corresponding to MT. The almost orthogonal isoeccentricity and isopolar angle contours of MT show that it contains a very orderly, almost conformal (angle-preserving) nonmirror image map of the contralateral visual hemifield (blue–purple). The electrophysiological border of MT as defined by visual field sign matches up quite closely (∼0.2 mm) with the myeloarchitectonic border of MT (thick continuous line). Slightly discrepancies might be due to difficulties in recovering the MT border from serial sections because portions of DLa/MTc can be myelinated as densely as MT itself (see Fig. 9, and Kaas & Morel, 1993, their Fig. 2).

The area between V2 and MT (e.g., along black-and-white dashed line) is now divided into 3 strips of alternating visual field sign. V2 is visible at the posterior end of the exposure as a thin strip of nonmirror image cortex (blue–purple) whose anterior border coincides with the myeloarchitectonically defined anterior border of V2 (thick continuous black line). Just in front of V2 is mirror image (yellow) DLp. DLp appears separated into two patches. The smaller more medial DLp patch is generally more eccentric than the more lateral patch; however, a small amount of the visual field is *re*-represented in the lateral patch (see contours and the arrow diagram). Moving anterior to DLp, there is a continuous nonmirror image (blue–purple) area, DLi. DLi appears to fuse with MT near the center of gaze representation of both of those areas. Adjoining DLi anteriorly is mirror image (yellow) DLa/MTc. A discontinuous piece of DLa/MTc appears to wrap at least part of the way around lateral upper field MT. The visual field representation here, about halfway around the lateral border of MT, is quite distorted (almost parallel isoeccentricity and isopolar angle contours; ambiguous/gray field sign). The posterior part of this region—marked by a small unlabeled tongue of nonmirror image (blue–purple) field sign that protrudes from MT+ laterally—corresponds to the vertical meridian representation (the DLa/FSTd border) that extends at right angles from the lateral border of MT mentioned above. The field sign pattern suggests that DLa may separate from MT at this point and continue laterally for a short distance. Some of the stained flatmounts in Tootell et al. (1985) and injection cases in Kaas and Morel (1993) are consistent with this notion.

Returning to our posterior-to-anterior traverse, we had already crossed V2- (nonmirror), DLp- (mirror), DLi- (nonmirror), and DLa/MTc- (mirror). The pattern of alternating visual field sign continues with MT- and MT+ (nonmirror), FSTd+ and FSTd- (mirror), and finally FSTv- (nonmirror). The field sign pattern in FSTd- and FSTv+ is a little noisy, possibly the result of undersampling these small areas.

The region lateral to the “DLi+” label is near the expected border with VP and VA. It was difficult to decide where to draw the boundary between VA and DLi (both nonmirror), and DLp and VP (both mirror). Also, there was a small gap in one of the penetrations rows (just lateral to the lateralmost “DLp-” label). There is some evidence that upper field VA and VP may both have small *lower* field representations that are not part of DLp and DLi (case 4 and unpublished chronic recording data). This would require that receptive fields cross and recross the horizontal meridian passing from DLp into VP, and DLi into VA. There were, in fact, a few alternating lower and upper field points consistent with such a double reversal (visible in arrow diagram in Fig. 5 just lateral to the lateralmost DLp-border but smoothed over in Fig. 7 due to many nearby lower field points). Newsome and Allman (1980) showed that further laterally (ventrally), the border between VA and VP is marked by a clear upper field vertical meridian representation that is correlated with a prominent band of callosal connections. A lower field vertical meridian representation is visible in the polar angle plot in Fig. 7 at the lateral posterior corner of the craniotomy. We have provisionally labeled this region “[VP]” and “[VA]”.

We now turn to areas lateral to MT. Directly lateral to upper field DLa/MTc is inferotemporal cortex. The field sign picture suggests that it may be divided into a posterior mirror image (yellow) part, ITc, and an intermediate nonmirror image (blue–purple) part, ITi. Except for a number of large diameter receptive fields coincident with the vertical meridian representation that emerges perpendicular to the lateral MT border, the receptive fields in ITc, as well as those in ITi, were quite small. Receptive field size increases toward the anterior lateral corner of the craniotomy in an unlabeled region that may represent a subdivision of ITi or the posterior border of ITr.

Medial and anteromedial to MT, three upper/lower field pairs with alternating visual field sign are encountered as one moves in a clockwise direction around MT: TP- and TP+ (mirror), TA- and TA+ (nonmirror), and MSTd- and MSTd+ (mirror). This region is heavily myelinated, though less so than MT (see also Figs. 3 and 9). There are a limited number of points supporting the existence of a fourth lower field representation in MSTv- (nonmirror).

Finally, back at the posteromedial corner of the craniotomy, there is evidence for a small nonmirror image area (blue–purple), DI, directly adjoining lower field V2, which is also nonmirror image. This pattern of receptive fields continuing into the upper field beyond the border of V2 without changing field sign (that is, unlike a mirror image V3d) was replicated in other cases where this area was explored in detail (see Figs. 12 and 16).

### Case 2—dorsolateral cortex (548 sites)

The craniotomy in case 2 was situated somewhat medial (dorsal) to the one in case 1 (see Fig. 3 for the approximate overlap between cases 1 and 2); case 1 was centered on lateral upper field MT while in case 2, only the medial border of MT was exposed. Case 2 was introduced in Sereno et al. (1994). The presentation here includes many not previously illustrated points from the superior temporal sulcus. A deformable template-based algorithm was used to nonlinearly warp the *x*–*y* locations from the penetration photograph into exact alignment with the flatmount using a set of eight marker lesions across the recording sites (see Sereno et al., 1994 for algorithm and example).

#### Flatmounted myeloarchitecture (Fig. 9, right)

A drawing of the pattern of myelination throughout all layers of the cortex (see Materials and methods) serves as the background for Fig. 9. All responsive penetrations as well as field sign reversals (dashed lines) have been superimposed on the myelin pattern. The marker lesions themselves are visible in the myelin drawing. As before, labels have been placed in exactly corresponding places in successive illustrations of different aspects of the data.

MT stands out as a densely myelinated region that occupies most but not all of a somewhat larger teardrop-shaped region (with the point of the teardrop at about 2 o'clock). This teardrop shape is also apparent in some of the cytochrome oxidase-stained flatmounts in Tootell et al. (1985; see, for example, their Figs. 1, 2, and 8). The anterior border of MT coincides with a slight drop in myelination before reaching the point of the teardrop (near marker lesion visible just above the “M” in “MSTd-”). The variations in the density of myelination within different parts of MT shown in Fig. 9 were consistent across many sections. They could therefore not be due to vagaries of the Gallyas stain in particular sections, though it is possible that they could be attributed to local differences in tissue fixation. The fact that a similar pattern of regional variation appears in MT in other cases argues against this last possible artifact. There are three main peaks in the myelin density within MT in Fig. 9—one in the posterior part of MT near the center of gaze, one under the “MT+” label in upper field MT, and a third under the anterior “MT-” label in lower field MT. Three similar maxima are visible in Fig. 3. The reduction in myelination density as MT dips into the superior temporal sulcus—previously shown in the sagittal section in Fig. 4A—is visible 1–2 mm above the “MT+” label in the Fig. 9 flatmount, and is not an artifact of the unfolding of the sulcus (it is also clearly visible just under the “MT” label in Fig. 3).

The border of MT is slightly blurry, surrounded by a thin less densely myelinated halo about 500 microns wide. The MT border may actually correspond to the inner boundary of the halo. There are several larger blob-like myelinated regions extending from the medial border of MT that make up part of DLa/MTc. Medial to the blobs is a relatively lightly myelinated region, DLi. Posterior and medial to DLi is a wide, somewhat more densely myelinated band corresponding to DLp. Yet further medially and posteriorly is a large, very densely myelinated region adjoining V2 that corresponds to areas DI and DM (there is a small poorly myelinated patch within DI). The density of patchy myelination in DM is equaled only by that in MT. Anterior to the most densely myelinated region (DM) there is a moderately myelinated area, VPP. The myelin density is further reduced just before reaching the posterior extremity of the Sylvian sulcus in PP.

V2 shows evidence of stripes. Previous work by Krubitzer and Kaas (1989, 1990) suggested that the myelin-dense stripes correspond approximately to the interstripe regions (pale stripes) in a cytochrome oxidase stain (this is analogous to V1 where the myelin dense matrix corresponds to the cytochrome pale interblobs). The myelin stripes in V2 were much more visible in dorsal V2 than in ventral V2. The anterior border of V2 is difficult to make out in a myelin stain in the cortex on the medial wall (immediately right of the top middle inset box in Fig. 9).

Anterior to the DL region just lateral to the end of the Sylvian sulcus there are a series of small myelinated patches that run approximately parallel to the Sylvian, and then turn slightly posteriorly to touch the anterior end of MT (labeled TD, TA, MSTd). Between DLa/MTc and TA, there is a lightly myelinated region, TP. Continuing clockwise around MT, on the lower bank of the superior temporal sulcus, is moderately myelinated FSTd. Finally, at the bottom left of the drawing there is a densely myelinated patch between the posteriormost tip of MT and V2 (unlabeled). This last region is not currently thought to be a distinct area. Several recording sites in this area in case 1 produced receptive fields near the center of gaze. A similarly located densely myelinated region is visible anterior to the “V2+” label in Fig. 13.

#### Receptive fields from DM, DI, DLp, DLi, and DLa/MTc (Fig. 9, left)

We have selected about 60 receptive fields arranged in four penetration rows (large dots connected by lines in right side of Fig. 9) that extend from DM (at the medial edge of the craniotomy) to MT (at the lateral anterior border) to illustrate the complex retinotopy in this region. These tracks were made by resampling the anterior–posterior penetration rows. Like the two tracks illustrated in case 1, these four resampled tracks are oriented approximately perpendicular to the MT border but are rotated in a clockwise direction compared to the those in case 1; the receptive fields in these tracks are more eccentric than those from the case 1 tracks.

The left side of Fig. 9 shows receptive fields for the four rows of penetrations across five areas: DM, DI, DLp, DLi, and DLa/MTc. The receptive field centers for all 500+ penetrations are shown at the lower middle. In the five receptive field plots, the shading of the receptive fields (4 levels) indicates the anteroposterior position of the track; lightest is posterior, and darkest is anterior. The order of the penetrations within a row is then indicated by receptive field overlapping—the arrows indicate the first (most medial) receptive field in each row, while successive receptive fields occlude those from earlier in the row.

The organization of DM differs from all the other areas in that the most posterior row in the cortex resulted in the most eccentric receptive fields (light gray receptive fields are peripheral); all the remaining areas show the opposite pattern where the most posterior rows resulted in receptive fields nearest the center of gaze (light gray receptive fields are central). This shift creates a particularly complex pattern of retinotopy in the region surrounding DM. As can be seen in the receptive field plots for DM, receptive fields begin near the lower field vertical meridian and approach the upper field vertical meridian. As the tracks pass into DI (nonmirror image), the visual field sign reverses partway into the upper quadrant without individual receptive field tracks turning back on themselves; the receptive fields continue toward the vertical meridian, but now with the posterior row (lightest) nearest the center of gaze. This field sign reversal can be visualized by considering that lines connecting each row of receptive fields all have to cross each other going from DM+ to DI+.

The remaining three areas show the more conventional pattern where visual field sign reverses near a meridian. On passing into DLp (mirror image) from DI (nonmirror image), receptive fields simply reverse at the upper field vertical meridian. They progress almost to the lower field vertical meridian where they reverse again upon passing into DLi (mirror image). In DLi, receptive fields approach the horizontal meridian. A final reversal occurs at the horizontal meridian upon passing into DLa/MTc (mirror image) where receptive fields return once more to the lower field vertical meridian (the border of MT). This case provides evidence for a small *upper* field representation in DLp. Receptive fields moved very rapidly into the lower field (curved arrows in DLp), however, sometimes without overlapping. In extensive experience with tangential penetrations through extrastriate cortex in chronically recorded cases, we have rarely come across a true discontinuity in a receptive field sequence. This may be one; but it is still possible we undersampled the rapidly changing topography here.

The receptive field plots show that much of the upper quadrant is re-represented in DM, DI, and DLp, while much of the lower quadrant is re-represented in DM, DLp, DLi, and DLa/MTc. This fact is not as immediately apparent in our contour plots because the center of the receptive fields usually does not reach the meridians, and because there is some unavoidable smoothing of maxima and minima with any reasonably stiff interpolation. In many respects, however, the contour plots are to be preferred since they quantitatively take all the data into consideration.

#### Isoeccentricity and isopolar angle maps (Figs. 10 and 11)

**Fig. 10.**
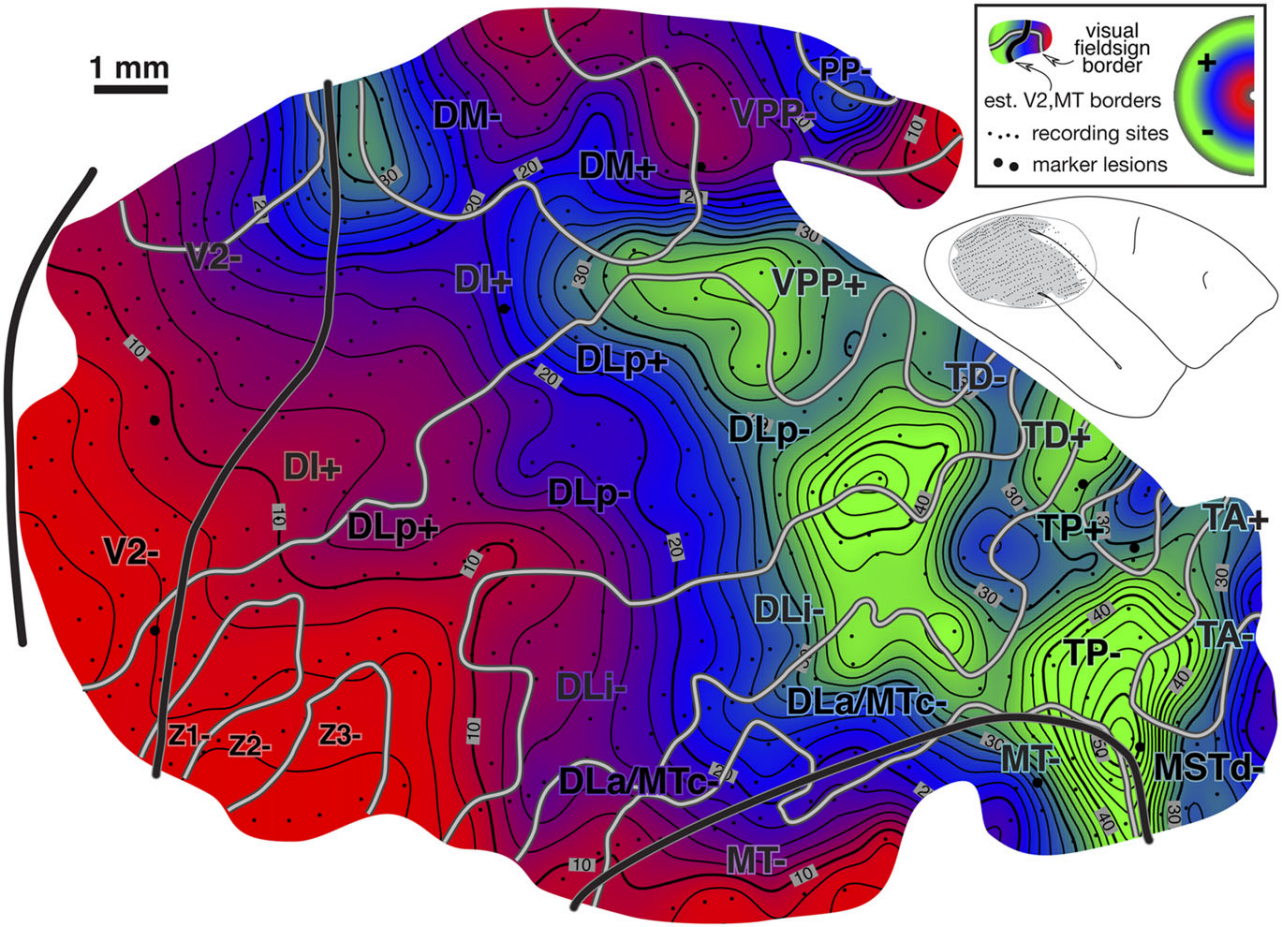
Cortical map of receptive field *eccentricity* for case 2. The contour map was shaded as before (central eccentricities are red, middle eccentricities blue, and peripheral ones green). There is a large center of gaze representation at the lower left which has two protrusions anteriorly in DLp and a smaller one in DI. There are several returns to more central fields including at the DM/VVP border, then further anterior in PP, at the anterior and medial edge of TP, and beyond the extreme periphery of MT in MSTd. In V2, isoeccentricity lines are markedly tilted moving into the periphery forming the beginning of a saddle point. There are 4 small peripheral maxima. The first is in lower field VVP. The second spreads across the peripheries of DLp, DLi, and DLa/MTc. The third is in TD, and the fourth is at the boundary between MT and MSTd.

**Fig. 11.**
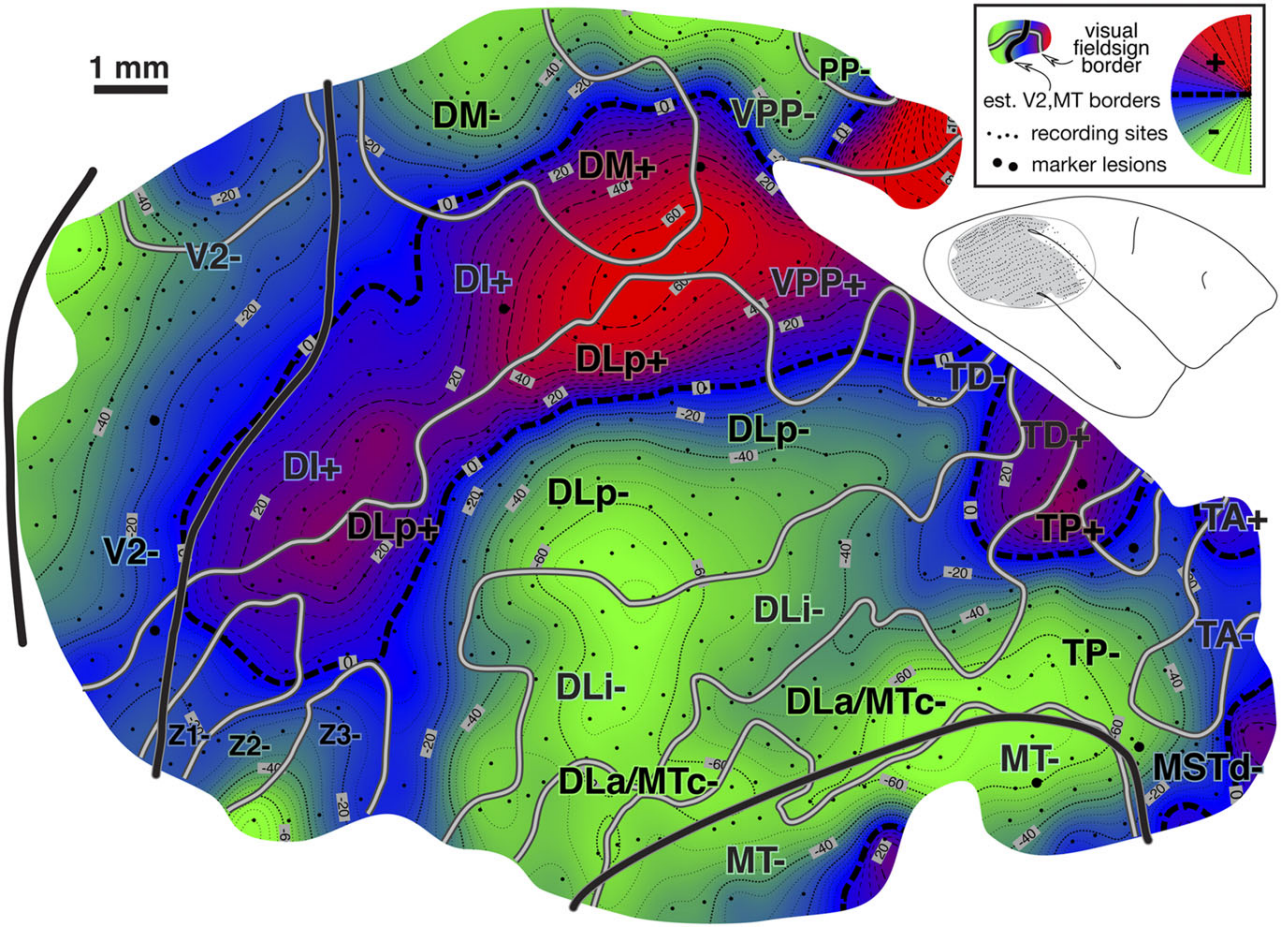
Cortical map of receptive field *polar angle* for case 2. The contour map was shaded as before (lower field is green, horizontal meridian blue, and upper field is red). This case clearly shows that upper-field-only DI directly contacts lower field V2 without any V3-like reversal toward the lower field for more than 2 mm along the V2 border. Moving anterior and laterally from there, receptive fields rapidly move back into the lower field in DLp. The horizontal meridian reversal (bluish) at the border between DLi and DLa/MTc is hard to see because the edge of rather large receptive fields reaches the horizontal meridian but their centers do not. Finally, upper fields return far anteriorly in PP, at the TP/TD border, and in MSTd (which was more fully explored in case 1).

Interpolated contour plots of receptive field eccentricity and polar angle for case 2 are shown in Figs. 10 and 11. Visual field sign transitions as well as the estimated myeloarchitectonic border of V2 and MT are marked on the shaded contour maps as before.

The eccentricity map in Fig. 10 shows a similar pattern to case 1 in the region surrounding MT, where eccentricities generally increase moving anteriorly (red/blue/green). Beyond the anterior border of MT, eccentricities move more centrally in both cases (slightly stronger in case 2, in both cases near the label “TA-”). In both cases, there is also a small posteriorly-directed protrusion of intermediate eccentricities just medial (dorsal) to MT (at bottom Fig. 10 near “DLa/MTc” label and at the top of Fig. 6 near the same label). Further medially, in a region not covered in case 1, several more large near-center-of-gaze eccentricity minima (red) appear at the boundary between DM and VPP.

The case 2 polar angle map in Fig. 11 shows that upper field-only DI directly contacts lower field V2 without any V3-like reversal toward the lower field for almost 2 mm parallel and perpendicular to the V2 border (see below). Anterior from this point, upper visual fields (red) reverse in DLp toward the horizontal meridian and go back into the lower field meeting DLi. Further anteriorly, there are two upper field vertical meridians (red), one at the border between TD and TP, and a second barely visible at the edge of the map in MSTd- (fully explored in case 1).

#### Visual field sign map (Fig. 12)

**Fig. 12.**
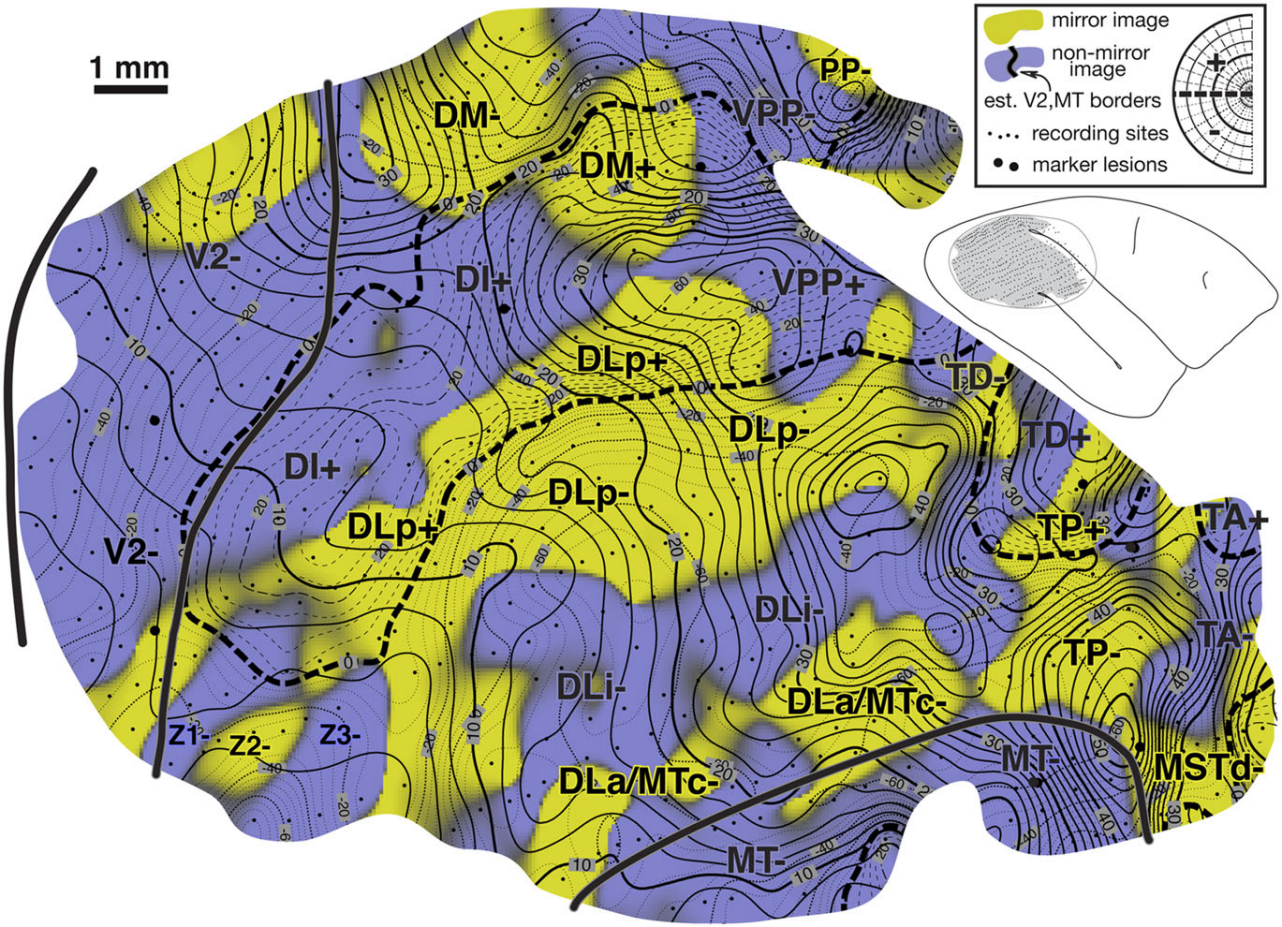
Cortical map of *visual field sign* for case 2. The conventions are the same as in Fig. 8. A larger portion of V2 was exposed (nonmirror/blue–purple). Nonmirror DI (yellow) directly adjoins the anterior border of V2 with the same visual field sign as V2. DLp (mirror/yellow), DLi (nonmirror/blue–purple), and DLa/MTc (mirror/yellow) form three sinuous strips of alternating field sign between DI and MT; MT is visible at the lower right corner as a small patch of nearly orthogonal isoeccentricity and isopolar angle contours. This case provided more detailed evidence for area TP (mirror/yellow) and area TA (nonmirror/blue–purple). The visual field sign picture was somewhat noisy in the region of area TD, probably because we did not sample the rapidly changing retinotopy in this small area densely enough. Near the medial edge of the exposure (top), lower field DM is visible as a prominent mirror image patch adjoining V2. Its upper field representation unexpectedly turned anteriorly, away from V2. Anterior to DM is nonmirror VPP. There appeared to be another area beyond VPP, labeled PP-. Finally, there were several field sign reversals near the center of gaze (Z1, Z2, Z3) in a region not previously thought to contain multiple areas.

A complete visual field sign map for case 2 taking all receptive fields into account is shown in Fig. 12. Lower field V2 makes up the largest single patch of nonmirror image field sign (blue–purple) at the posterior extent of the craniotomy (save for a small patch lined up with a pair myelinated stripes). The anterior myeloarchitectonic border of dorsal V2 corresponded approximately with the horizontal meridian. But then receptive fields anterior to V2 simply continued into the upper field *without* reversing visual field sign (staying purple–blue). This is consistent with the previous discussion of the receptive field transition between DM and DI, where a field sign reversal *does* occur, since DM is mirror image (yellow). We labeled this region DI. It contains only an upper field representation. This pattern had already been observed in case 1 (upper left of Fig. 8) and was confirmed in the succeeding case (see Fig. 16).

Further medially along the V2-/DM-border, there is in fact a clear visual field sign reversal. However, this change was primarily the result of *eccentricity* coming to a peak and then falling off again (approximately at the 30 deg isoeccentricity contour); isopolar angle contours continued into lower field DM without much disturbance/reversal. This contrasts with a “normal” horizontal or vertical meridian field sign reversal where isoeccentricity contours continue approximately straight into the adjoining area and isopolar angle contours double back. The visuotopy reported here inside lower field DM is quite similar to that initially described by Allman and Kaas (1975) but the map is rotated and distorted when compared to their standard schematic summary diagram. Lower field DM is the most strongly myelinated area in this region of cortex (Fig. 9, right).

Adjoining lower field DM *anteriorly* (as opposed to laterally) is a slightly less densely myelinated region of upper visual field with the same field sign as lower field DM (mirror image). We labeled this area “DM+”. It seems likely that previous studies based mainly on myeloarchitecture may have instead (or in addition) included the medial parts our upper field DI (the nonmirror image region under the more medial “DI+” label) in upper field DM (see Discussion for alternate parcellations).

Directly anterior to upper field DM (as we have defined it) is a nonmirror image area containing a distorted representation of most of the visual field labeled VPP. Like DM, this area has a more medially situated lower field representation and a more lateral and anterior upper field representation. A lesion placed at the physiological DM/VPP border revealed almost no change in myelination upon entering upper field VPP from upper field DM (see lesion at anterior medial corner of craniotomy in Fig. 9, right). These two areas are joined at the center of gaze and share an approximate vertical meridian in both their upper and lower visual fields. There was limited evidence in this case for yet another reversal in field sign beyond VPP in PP (mirror image). This was confirmed in the following case.

Moving laterally, the retinotopy of the DL region is quite complex and can be difficult to appreciate in single tracks across this region because of receptive field scatter. The field sign technique nevertheless clearly reveals three parallel strip-like areas in between DI/DM and MT. Immediately lateral and anterior to DI is DLp (mirror image, yellow). It consists of a thin elongated upper field representation adjoining DI and a wider lower field representation whose anterior border is shared with DLi. Nonmirror image DLi (blue–purple) occupies a thin sinuous region a little over 1 mm wide containing a lower field representation. The craniotomy did not extend far enough laterally in this case to determine whether or not there was also an upper field part of DLi (there was only a minimal upper field DLi in the previous case). DLa/MTc appears as a third even thinner strip of lower field (mirror image, yellow) representation between DLi and MT. DLa/MTc as defined electrophysiologically seems to be wider than the very thin myelinated band around MT. There was a tendency for the field sign borders to follow the myelinated blobs (see Fig. 9, right).

The retinotopic organization of the cortex anterior to the greater DL/MT region is even more complex. Moving anteriorly in lower field DLa/MTc, eccentricity increases until it reaches a peak at the end of MT (near the most anterior lateral lesion). Moving medially from that point, receptive fields return toward the center of gaze in a lightly myelinated region labeled TP. TP (mirror image) has the same field sign as DLa/MTc and is distinguished from anterior DLa/MTc by the fact that it contains a re-representation of much of the same parts of the lower visual field represented in DLa/MTc. Receptive fields eventually cross the horizontal meridian into the upper visual field, suggesting that TP has a small upper field representation as well. Continuing further medially, visual field sign reverses once again in the upper field in a more densely myelinated area labeled TD (nonmirror image). Yet further medially, receptive fields cross back into the lower visual field (near the “TD-” label). The pattern of visual field sign in the region near TD is somewhat noisy; even denser sampling would be required to make definitive statements about retinotopy there.

Returning to lightly myelinated mirror image TP (yellow) and now moving laterally and anteriorly, visual field sign reverses in a more densely myelinated region labeled TA containing mostly a nonmirror image (blue–purple) lower field representation. Several recording sites suggest that TA may have a medial upper field representation. Yet further laterally, directly anterior to MT, there is another reversal of field sign passing into MSTd (mirror image) from either MT or TA (both nonmirror image). Despite the small size of these areas relative to the sampling density, they agree surprisingly well with the map of this region presented for the previous case (Fig. 8). The border between MT and MSTd is marked by a small stripe of less dense myelination (Fig. 9, right).

Finally, at the posterior lateral border of the craniotomy, there are four stripes of alternating field sign near the center of gaze representation of V2. The most posterior (unlabeled) mirror image stripe is actually within V2 and corresponds roughly with a myelinated band. The three more anterior stripes labeled Z1, Z2, and Z3 showed an alternation in field sign supported by a reasonable number of recording sites. This region is near the center of gaze, however, where receptive field sequences can be difficult to make out, and merits a closer examination in the future with a higher sampling density to determine whether Z1, Z2, and Z3 actually constitute distinct areas.

### Case 3—dorsal cortex (549 sites)

A large craniotomy was made in case 3. All the recording sites, however, were concentrated in the medial part of the exposure. This case overlaps with the medial parts of case 2 but is almost entirely medial to case 1 (see Fig. 3 for approximate case 1–3 overlap). As with case 2, a nonlinear deformable template algorithm was used to warp the *x*–*y* locations from the penetration photograph into exact alignment with the stained flatmount using a set of nine marker lesions.

#### Myeloarchitecture (Fig. 3 and 13)

**Fig. 13.**
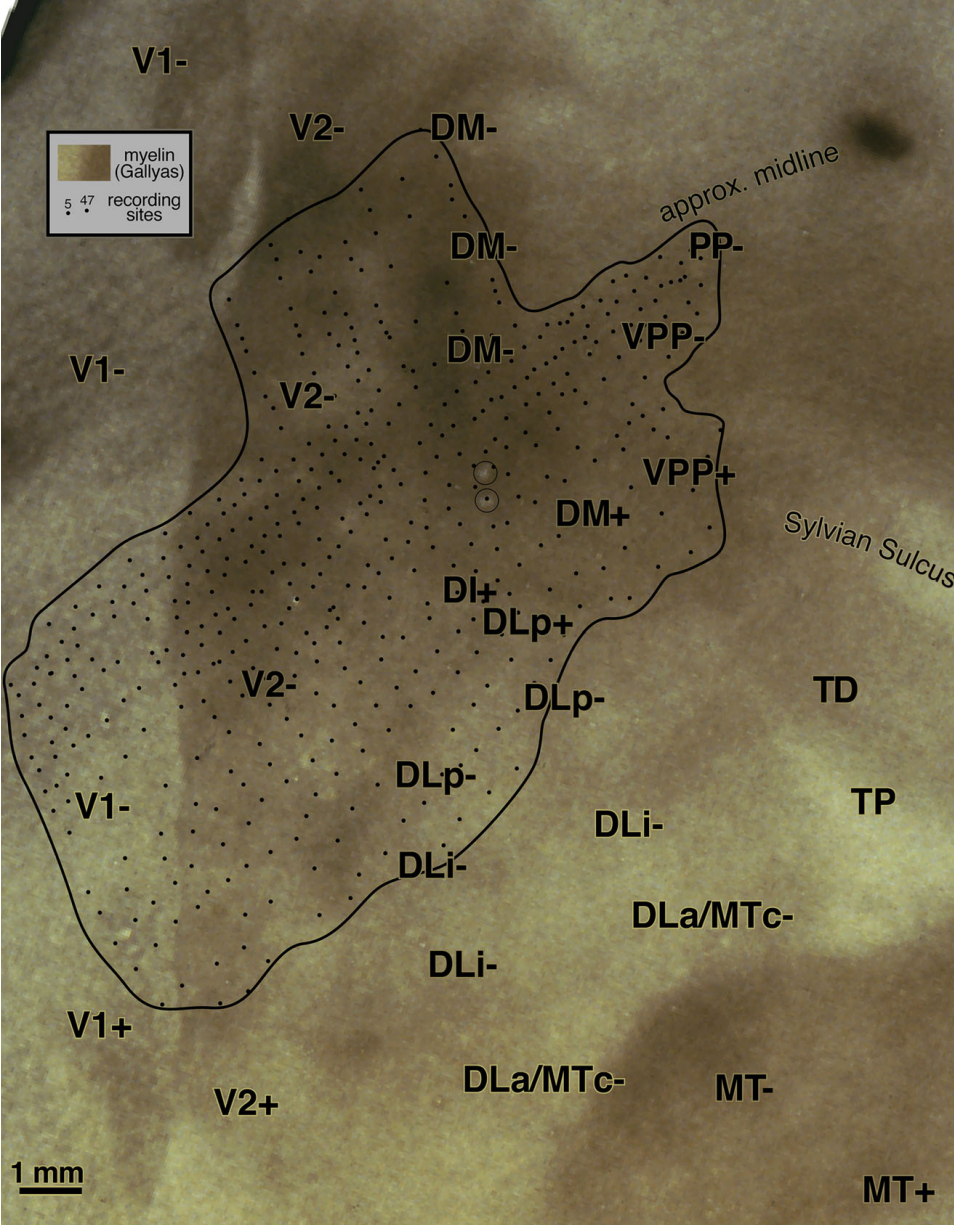
Location of all recording sites for case 3 superimposed on single myelin-stained flatmount section. The V1 and the MT borders are the most obvious. The mottling pattern in V1 is probably due to increased myelination of interblobs. The anterior border of V2 is difficult to see. It was moderately clearer—as were V2 stripes and other features—after collapsing across sections (see text for description of myelin features here and in stacked drawing). A pair of marker lesions are indicated by two open circles. They were placed just anterior to the anterior horizontal meridian border of V2. The density of myelination here was similar on the V2 side and the DM side of the border. This is a close-up of the section in Fig. 3.

The pattern of myelination proved to be remarkably robust to these long experiments. Many fine details could still be resolved in flatmounts even after 100 h of recording. Fig. 13 shows a close-up image of a single myelin-stained flatmounted section from case 3 with all recording locations. The image approximately covers the area of the craniotomy. Fig. 3 shows a more sparsely annotated lower magnification view that includes most of the neocortex as well as the approximate boundaries (dashed lines) of the sets of recording sites in cases 1 and 2. The description below also relies on a composite drawing (not shown) made by stacking and superimposing all the sections to ameliorate variable plane-of-section artifacts. The illustrated section cuts approximately through layer 4, which was also the target depth of our recordings and marker lesions.

The sharpness of the V1 border stands out over others. It is slightly undulatory and has a characteristic anterior protrusion that has been identified as the V1 center of gaze representation (near “V1” label in Fig. 3, near “1 mm” label in Fig. 13). V1 has a mottled appearance. Sections in slightly deeper cortical laminae (not shown) even more clearly showed lightly myelinated roughly circular patches likely corresponding to cytochrome oxidase blobs surrounded by a more densely myelinated matrix that likely corresponds to interblobs (see Krubitzer & Kaas, 1989, 1990).

MT is the second most prominent feature in visual cortex, with a marked variation in density of myelination in different subregions that extended through many sections (Kaas & Morel, 1993). The posterior medial border of MT is the most distinct and most densely myelinated. As MT descends anteriorly into the superior temporal sulcus there is a sudden drop off in myelination density in peripheral MT originally recognized by Allman and Kaas (1971*a*), visible as a thin arc of slightly less dense myelination immediately below the “MT” label in Fig. 3 (and in Fig. 4A just above the “STS” label).

V2, like MT, shows a substantial amount of local variation in myelin density within its borders, with some evidence of stripes (clearer after superimposing all sections), but also larger scale variations; the medial and lateral periphery are more densely myelinated than central V2 (far laterally, the flattening was imperfect; two small horizontal ridges in the cortex in V2 that partly extend into adjoining areas resulted in inconsistent sampling of cortical lamina there). The V2 border is most apparent just medial to (up from) the center of gaze.

Further medially, densely myelinated area DM, as originally described by Allman and Kaas (1975), directly adjoins the anterior border of V2. The myeloarchitectonic border between DM and V2 was difficult to distinguish there. A closely-spaced pair of lesions placed visible in Fig. 3 just past the physiologically-defined V2 border (two open circles halfway between “DI+” and “DM-” labels in Fig. 13) sits in the middle of a small darkly myelinated region that extends out from V2 into DM. The densest core of myelination in DM near the double lesion corresponds to the near lower field DM. The density of myelination just beyond V2 falls off smoothly moving medially along the V2 border onto the medial wall. Anterior to the densest core of DM, there was a lightly myelinated boundary and then an almost as densely labeled region, VPP, whose upper field was situated just posterior to the end of the Sylvian sulcus. There was another anterior-posteriorly elongated myelinated patch anterior to VPP that continued along the lower bank of the intraparietal crease. At least the posterior part of this region corresponds to PP (originally named by Allman & Kaas, 1975). The cortex tends to be stretched more at the fundi of opened sulci such as the intraparietal crease, which may decrease myelination density; but it is also the case that the cortex is generally more lightly myelinated in sulci (Sereno et al., 2012).

Moving the opposite direction (laterally) from the densely myelinated DM core, there was a reduction in myelin density in DI and DLp. It was difficult to positively identify the anterior border of V2 in this region as well on myeloarchitecture alone, even after including all the sections. DI in particular appeared to fuse posteriorly with the myelinated V2 stripes. Lateral to DLp there was a very lightly myelinated region likely to be DLi. Just lateral to presumptive DLi, there was a large somewhat indistinct patch of myelination that extended from the medial border of MT almost to V2 (just above the posteriormost “DLa/MTc-” label and the “V2+” label in Fig. 13). Only the most anterior part of this patch corresponds to DLa/MTc. It is not certain what area occupies the posterior part of the patch, which touches V2.

Finally, we return to the areas surrounding MT (Figs. 3 and 13) (not recorded from in case 3). Starting at 12 o'clock, there is a triangular, lightly myelinated region adjoining the anterior medial border of MT that likely corresponds to TP. Just medial to this is a region of medium myelination probably corresponding to TD (its anteromedial border is formed by the lightly myelinated beginning of the opened Sylvian sulcus at the far middle right in Fig. 13). Continuing in a clockwise direction around MT, now looking at the anterior medial edge of MT in Fig. 3, there is a small “jet” of dark myelination extending a few millimeters anteriorly that probably corresponds to TA. Further lateral but still above the fundus of the STS is a larger moderately myelinated patch that probably corresponds to MSTd and MSTv. Continuing around MT to its lateral side, past the very light myelination in the fundus of the superior temporal sulcus and just lateral to the thin arc of reduced myelination within peripheral MT, there in a long triangular shaped patch of increased myelination on the lower bank of the STS that probably corresponds to FSTd and FSTv.

#### Isoeccentricity and isopolar angle maps (Figs. 14 and 15)

**Fig. 14.**
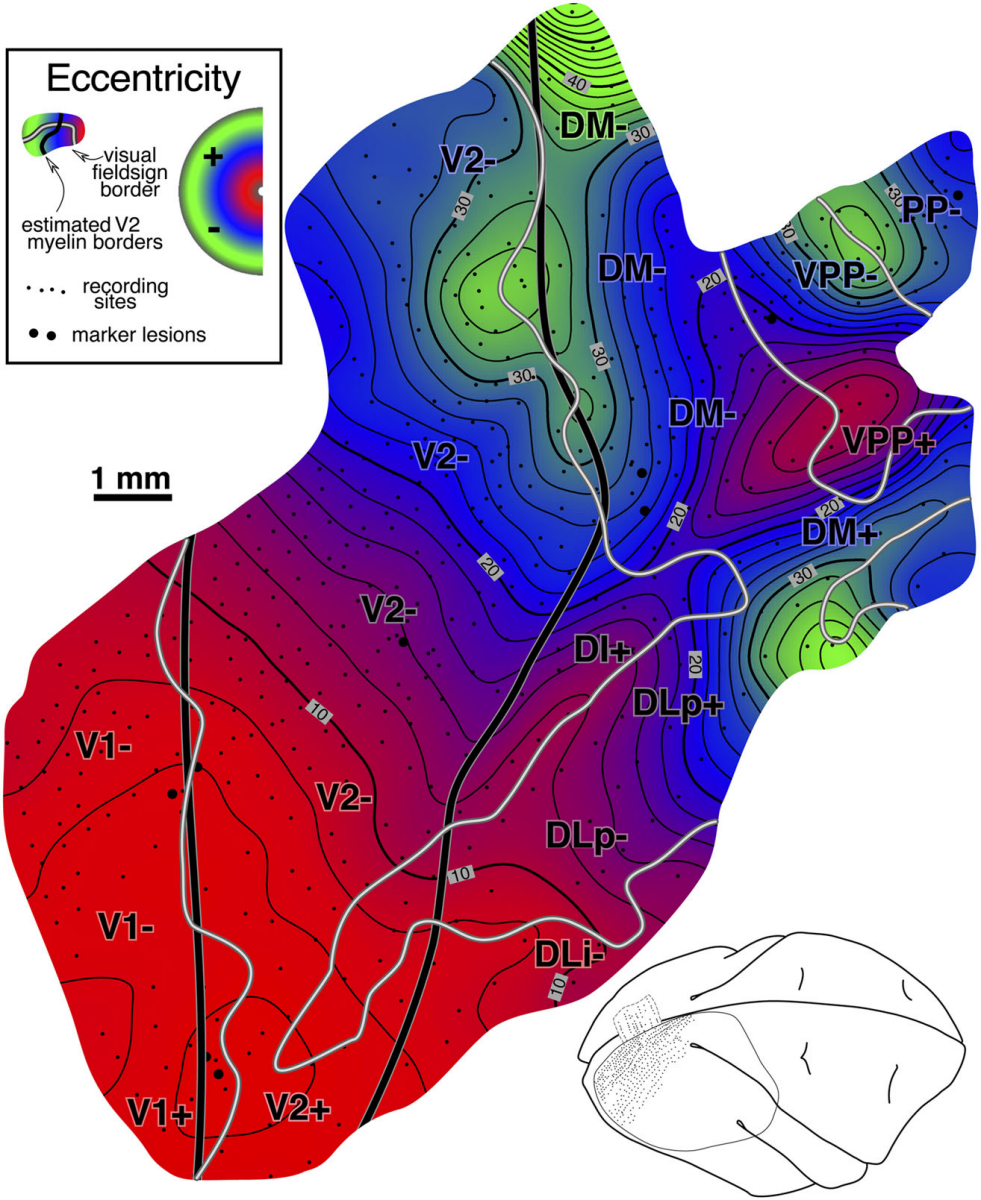
Cortical map of receptive field *eccentricity* for case 3. There are two main eccentricity minima (red)—one at the center of gaze of V1/V2 and another at the center of gaze of DM/VPP. Receptive fields are also approaching the center of gaze at the anterior border of PP and just beyond the lateral border of DM+. There were three main eccentricity maxima (green). The first was at the very top of the Figure at the V2/DM border from penetrations down the medial wall. Eccentricity is known to increase beyond that, further down the medial wall (Allman & Kaas, 1971*b*). There were two small but unexpected saddle points moving laterally (down) along the V2/DM border, just before the widest anterior–posterior extent of V2. The second main eccentricity maximum was at the VPP-/PP- border (top right) and the third at the DM+/DLp+ border. This last maximum was several mm anterior to the V2 border, suggesting that only the *lower* field of DM directly adjoins V2. As before, visual field sign borders traced from Fig. 16 are shown in gray. The lower right inset shows the location of the recording sites on the brain.

Fig. 14 shows the interpolated isoeccentricity map for case 3. As before, visual field sign transitions are marked (traced from Fig. 16).

**Fig. 15.**
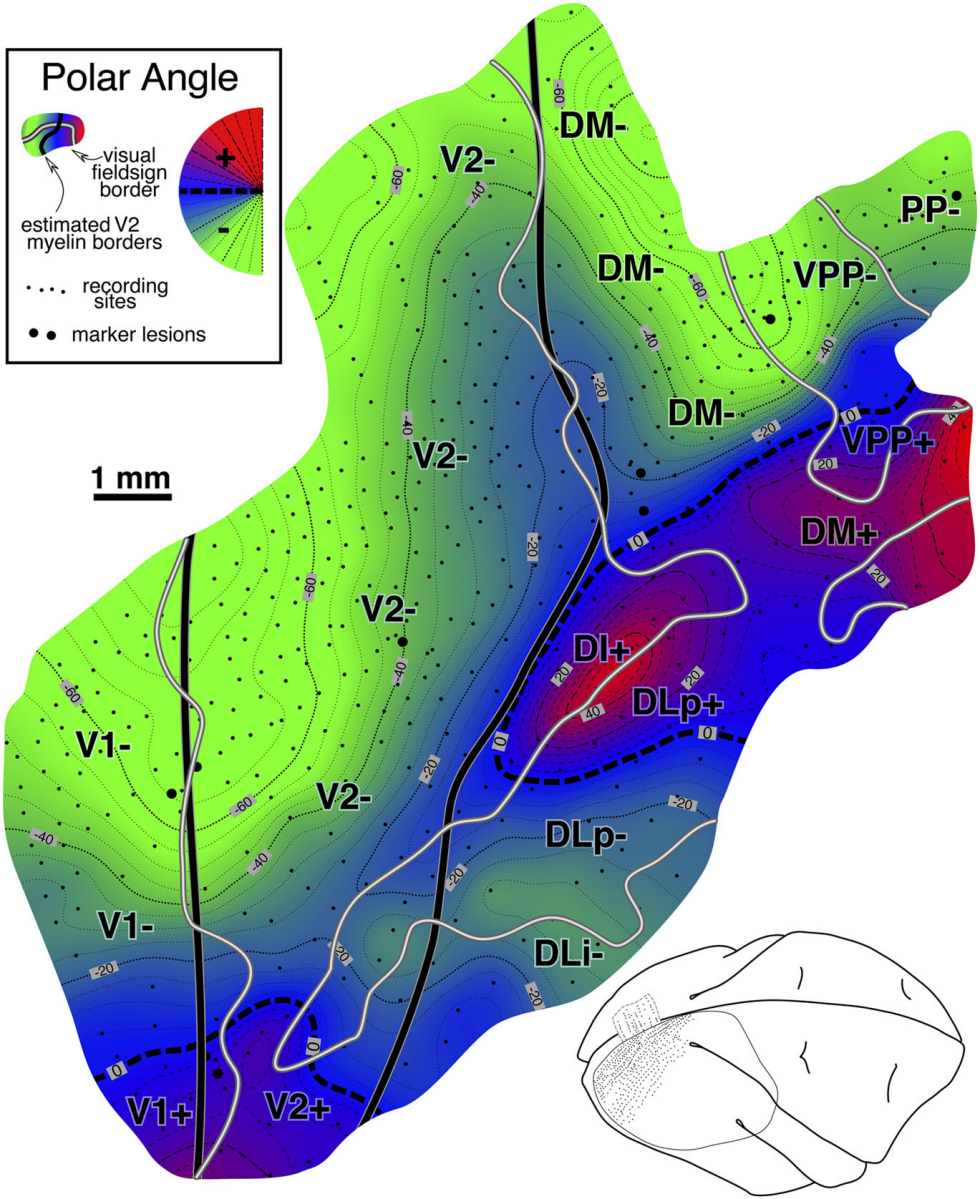
Cortical map of receptive field *polar angle* for case 3. The polar angle map shows a lower field vertical meridian (green) at the V1/V2 border, at the DM/VPP border, and at the anterior border of PP. The mediolateral lower field vertical meridian border (green) between DM and VPP bifurcates into anteriorly and posteriorly directed upper field vertical meridian branches (red) just past the center of gaze of DM/VPP. The posterior branch separates DI and DLp, while the anterior branch separates DM+ and VPP+. There are 4 re-representations of the upper field here.

**Fig. 16.**
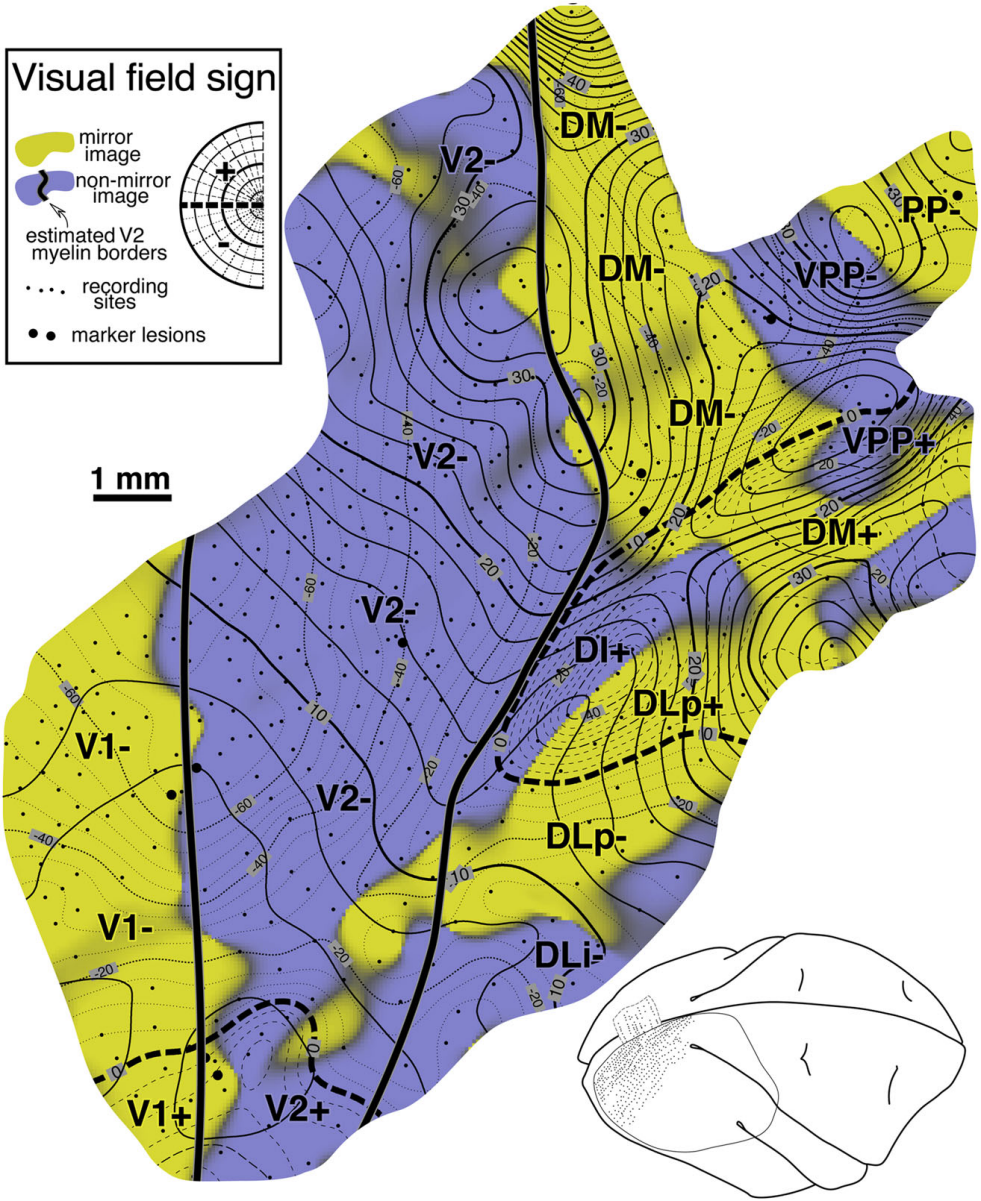
Cortical map of *visual field sign* for case 3. Areas V1, V2, DM, VPP, and PP appear as five mediolateral strips of alternating visual field sign across the top of the Figure. There is no visual field sign change as receptive fields anterior to lower field V2 move rapidly into the upper field in DI (middle of Figure). Just beyond this point, the anterior bend in the upper field representation of DM described in the previous case is again visible. The “finger” of upper field protruding posteriorly and laterally here into dorsomedial extrastriate cortex—which is otherwise predominantly characterized by lower field representations—was smaller here than in case 2. The sequence of reversals, however, was virtually identical, including a visual field sign reversal in the interior of the upper quadrant at the DI+/DM+ border like that illustrated in Fig. 9 and 12. V2 contained two small reversed sign patches, but showed little evidence of regular field sign reversals corresponding to stripes. V2 shows a marked anisotropic expansion in a direction parallel to the V1/V2 border. As before, isoeccentricity lines are more closely spaced than isopolar angle lines (2 deg *vs*. 5 deg).

The picture of eccentricity is characterized by two major eccentricity minima (bright red)—one at the shared center of gaze representation of V1 and V2, and a second at the shared center of gaze representation of DM and VPP near the anterior extremity of the recording area. Receptive fields were likely approaching two additional eccentricity minima: at the anterior medial edge of the recording area in PP, and just beyond upper field DM (immediately to the right of the “DM+” label).

There were three main maxima in eccentricity (green). The first was at the very top of the figure at the furthest medial recording site, which came from penetrations down the medial wall (see inset at bottom left) reaching almost 60 deg (but still not the end of V2). Moving along the V2 border from the center of gaze to the periphery, the regular march of contour lines is interrupted by two saddle points where the eccentricity gradient temporarily reverses just above the 30 deg contour. Interestingly, a nearly identical saddle point was uncovered in the previous case 2 in a similar position along the V2-/DM-border. In both cases, the increase in receptive field eccentricities temporarily flattens at a point just medial to the maximum anterior–posterior width of V2, just past the incongruent DI+/DM-border.

There was a second eccentricity maximum at the VPP-/PP- border (far upper right). Finally, there was a third maximum at the DM+/DLp+ border. This last maximum was several mm anterior to the V2 border, showing that only the *lower* field of DM directly adjoins V2.

Fig. 15 shows an isopolar angle map for case 3. There are four main *lower* field vertical meridian representations (green, polar angle minima). The first is along the V1-/V2- border. Anterior to this is a second lower field vertical meridian at the DM-/VPP- border; this extends onto the medial wall. Finally, receptive fields approach the lower field meridian for a third time in anterior PP. Finally, there is a near lower field vertical meridian representation at the DLp-/DLi- border, which corresponds with the medial end of the dense band of callosal connections discovered within greater DL by Cusick et al. (1984).

There are three main *upper* field vertical meridians in the map (red, polar angle maxima). The first was just visible at the lateralmost portion of the map along the upper field V1/V2 border (lower left). The second and third upper field meridians were notably *not* parallel to the second and third *lower* field meridians just described. The second upper field vertical meridian forms the DI+/DLp+ border, along the axis of the diagonal finger of upper field. This meridian is almost orthogonal to the long mediolateral axis of lower field DM. Notably, we found a handful of receptive fields with centers slightly into the ipsilateral field between the “DI+” and “DL+” labels. Anterior to that second meridian border is another wider polar angle maximum at the border between DM+ and VPP+. This border is elongated in an anteroposterior direction. It stands in contrast to the almost mediolateral border between DM- and VPP-. Starting at the DM-/VPP- border and moving laterally toward the DM/VPP center of gaze representation, the lower field vertical meridian (green) bifurcates into anterior and posterior branches as it continues into the upper field (red). This quite precisely recapitulated the situation observed in the previous case 2, although the upper field finger there was more ample.

#### Visual field sign map (Fig. 16)

Fig. 16 shows a visual field sign map made from the isopolar and isoeccentricity maps, which have both been included for reference. To a large extent, contours are locally orthogonal to the isoeccentricity contours, suggesting that the binary classification of the field sign angle, *λ*, produced by our coloring scheme has merit.

As expected, the V1/V2 border is marked by a mirror image (yellow) to nonmirror image (blue–purple) visual field sign transition. There are two small intrusions of mirror image cortex into V2—one near the center of gaze beginning just lateral to DI and another on the medial wall near the eccentricity saddle points at the V2-/DM-border. The more peripheral flip is due to the saddle point in eccentricity while the more central flip is due to a saddle point in polar angle associated with the DLp-/DLi- vertical meridian (the callosal band), which appears to protrude slightly into V2. Given the regularity of contour lines in other parts of V2 which also have stripes, these flips do not appear to be due to local duplication of the visual field within different stripe types.

Much of the medial extent of the anterior border of V2 is adjoined by lower field mirror image DM. Moving laterally in DM, just after passing the pair of lesions, however, the upper field of DM appears to turn anteriorly, away from the V2 border. Upper field DI is intercalated between V2 and DM here. A similar anterior bend in upper field DM (to make room for nonmirror image DI) was observed in case 2, although DI was much larger in case 2.

Lateral to DI is a small mirror image upper field representation, DLp+. The retinotopy is quite complex in this region. DLp+ and DM+ are both mirror image representations of much of the same part of the visual field; however, the center of gaze in DLp+ is posterior and lateral while the center of gaze in DM+ is anterior and medial. Their peripheral representations almost touch. Note, however, that since they have the same field sign, they cannot share a congruent peripheral border. The upper field vertical meridian, which forms the medial border of DLp+ does continue into DM+, with eccentricity coming to a peak at the DLp+/DM+ border. But the horizontal meridian of DLp+ is *laterally* situated while the horizontal meridian of DM+ is *medially* situated; schematically, DLp+ and DM+ only share a visual field corner.

VPP (nonmirror, blue–purple) and PP (mirror, yellow) stand out at the anteromedial extent of the recording area. VPP joins DM along a vertical meridian, while PP joins VPP along the periphery. The laterally situated upper field representation of VPP was rather small, and we only recorded lower field receptive fields in PP. However, it is likely, judging from the previous case, that we may have missed more upper field VPP and PP just anterior and lateral to the edge of the recording area in this case.

Returning to the DI+/DLp+ region, and moving laterally from DLp+, receptive fields cross into the DLp lower field representation—the first of the three lower field representations between V2 and MT revealed in the previous cases. Yet further laterally, there is the hint of a visual field sign reversal, probably corresponding to the beginning of lower field DLi. The lateral edge of the recording area (see Fig. 13), just barely made it to the lightly myelinated region between V2 and MT previously suggested to correspond to DLi.

The receptive field charts in the previous case (see Fig. 9) were used to illustrate a complex progression where visual field sign changed in the middle of the upper quadrant (at the DM/DI border). It is possible to make out an identical pattern in this case. Starting in lower field DM, the visual field sign is mirror image (yellow). Moving toward DI, receptive fields cross the horizontal meridian into the upper field. Before reaching the vertical meridian, the field sign changes to nonmirror (blue–purple) at the DM/DI border in the middle of the upper field, just as in the previous case. Receptive fields then continue toward the upper field vertical meridian, reaching it at the DI+/DLp+ border, where field sign changes back to mirror image (yellow) upon entering DLp+. The exact neighbor relations between areas are quite remarkably preserved despite the substantial differences in the overall size of the areas between the two cases; DI here is roughly one-quarter the surface area of DI in case 2.

By comparing the double contour map in Fig. 16 with the V2 myelin stripes from the stacked myelin drawing, the stripes were found to lie along lines in the visual field that are about halfway in between isoeccentricity and isopolar angle lines. Translated back into the visual field, V2 stripes would therefore lie along spirals.

It has been suggested that the visual field is re-represented within each stripe (Rosa et al., 1988). One possibility of how this might be arranged would be for visual field sign to alternate every other stripe. To examine this, we reduced the stiffness of our interpolated surface to make sure that we were not smoothing over such alternations. Although several small mirror image regions appeared, they did not seem to be correlated with the stripe pattern visible in the myelin stain (if the stiffness of the interpolation is too low, “tent”-like interpolated surfaces and artifactual field sign reversals will be generated even for noiseless data). Thus, although the visual field appears be re-represented in each kind of stripe (Rosa et al., 1988; Roe and T’so, 1995), our data are consistent with this occurring as a result of receptive field jitter as opposed to systematic mirror/nonmirror flips in field sign. Yet higher retinotopic sampling densities using optical recording techniques (see Discussion) could resolve this question.

By comparing the spacing between the isoeccentricity and isopolar angle contours, the substantial anisotropic stretching of the visual field map in V2 mentioned previously with respect to the receptive field plot becomes more apparent. Despite the fact that the isoeccentricity contours are spaced more closely in the visual field than the isopolar angle contours (there is a new contour every 2 deg of eccentricity *vs.* every 5 deg of polar angle), the isoeccentricity contours are more widely spaced than the isopolar angle contours on the cortex. This indicates an expansion of the V2 visual map (defined by receptive field centers) roughly in the direction of eccentricity in V2.

### Case 4—lateral cortex, cytochrome oxidase (244 sites)

#### Cytochrome oxidase pattern (Fig. 17, top)

**Fig. 17.**
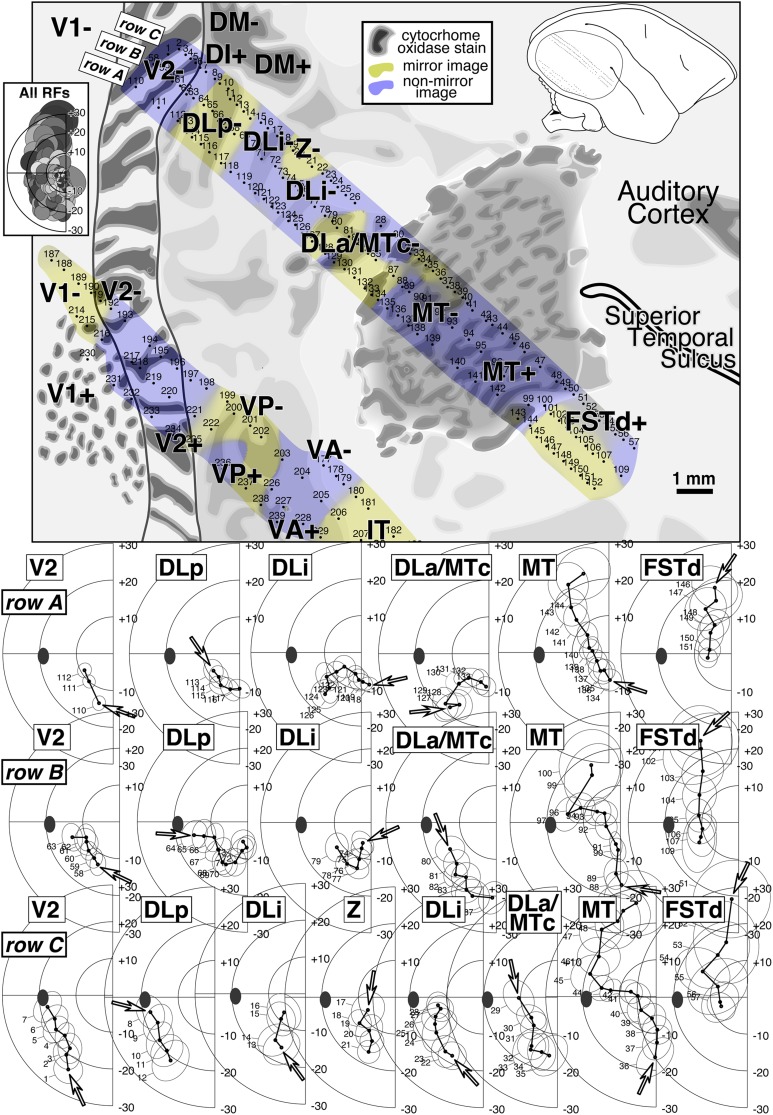
Case 4 cytochrome oxidase, penetrations, visual field sign (top) and receptive field plots (bottom) for retinotopic data in Fig. 18. A patchy, densely stained region adjoining V2 (top left), where V2 abruptly widens, likely corresponds to DM. Its lateral part, probably upper field DM, bends anteriorly away from V2. MT cytochrome oxidase patches were larger than V1 blobs but smaller than those in DLa/MTc. TP, which is lightly myelinated, appears as a cytochrome oxidase-dense triangle adjoining the anterior medial MT border. DLp and VP were more densely stained than DLi and VA. The transparent visual field sign overlays match architectonic borders to within our overlay accuracy limit (100–200 microns). A small finger of more dense staining extends into lightly stained DLi from the medial side overlapping uncertain area Z. Earlier cases suggest that upper field DI, DM and/or VPP (not lower fields) lie just medial to the recording area. Receptive fields for the three medial rows are illustrated below, where open arrows indicate receptive field from most posterior penetration in each area. Row C (1–57, bottom) showed seven vertical or horizontal meridian reversals. Receptive fields start near the lower field vertical meridian in V2, approach the horizontal meridian, return to the lower field vertical meridian in DLp, then reverse and approach the horizontal meridian again in DLi. After a small reversal in an area of uncertain identity (Z), receptive fields approach the horizontal meridian again, except at greater eccentricity; both vertical-to-horizontal meridian traverses were likely in area DLi, which has sinuous borders. Receptive fields then reverse at the horizontal meridian and return to the lower field vertical meridian in DLa/MTc, reverse again at the border of MT, and continue into the upper field. The final reversal at the upper field vertical meridian in FSTd coincides with a jump in receptive field size. Row B (58–109, one up from bottom) reversals closely follow the Row C sequence, except that the additional reversal and central deviation in DLi is less prominent (see last DLp receptive fields, first few in DLi). Receptive fields are less eccentric in Row B than in Row C. Row A (110–152, two up from bottom) sequences are similar to Row B, traversing V2, DLp, DLi, DLa/MTc, MT, and FSTd, and even less eccentric. The reversal at the border between DLi and DLa/MTc occurs some distance from the horizontal meridian suggesting complex retinotopy in the DLa/MTc “blobs” was undersampled.

The cytochrome oxidase staining pattern in case 4 was collapsed across all flatmounted sections using radial blood vessels and then schematized in the top of Fig. 17. A handful of V1 cytochrome oxidase blobs were drawn for size reference. Stripes were apparent in V2, but it was difficult to divide the darker stripes into two kinds (see case 5). As in cases 2 and 3, V2 bulges anteriorly at it approaches the dorsal convexity of the hemisphere. There are several very densely stained cytochrome oxidase patches extending anteriorly from the bulging part of V2 that likely correspond to DM and DI. Interestingly, the lateral border of the dark putative DM patch bends anteriorly, away from V2—a correlate of the proposed anterior bend in the upper field representation of DM. A similar anterior bend in a densely stained area adjoining dorsal V2 is beautifully illustrated in one of Shipp and Zeki's cytochrome oxidase-stained owl monkey flatmounts (Shipp & Zeki, 1989; their Fig. 11). Directly lateral to DM along the V2 border is a more moderately stained patch, DLp. Moving toward MT, the density of staining is further reduced in DLi. A region of very light staining formed a light horseshoe around the posterior half of MT (a similar pattern is visible in several flatmounted hemispheres in Tootell et al., 1985). This area was somewhat larger than DLi as retinotopically defined here.

It was difficult to precisely determine the border of MT on the basis of cytochrome oxidase staining alone. There was a large, densely stained MT core, marked by darkly stained patches that were several times the size of V1 cytochrome oxidase blobs (cf. Born & Tootell, 1992). The density of the cytochrome oxidase staining fell off gradually on reaching the border. The darkly stained patches were largest in the border region. There was a small, triangular region on the medial border of MT that was somewhat *more* densely stained for cytochrome oxidase than yet more medial cortex; this seems to correspond to the *lightly* myelinated area, TP. Past MT, there were several densely stained patches in FST. Finally, there were several patches of dense cytochrome oxidase staining along the lateral lip of the Sylvian sulcus (not shown—under the inset). Primary auditory cortex was visible at the far anterior extent of the flatmount.

#### Receptive fields from V2, DLp, DLi, DLa/MTc, MT, and FST (Fig. 17, bottom)

Receptive fields from the three long medial rows of penetrations (∼150 points total) are broken down by area. In this case, we made very closely spaced penetrations at the expense of covering a large area, in order to better demonstrate the degree of local orderliness in receptive field progressions. Because of the small area covered, some ambiguity remained about the identity of some of the regions traversed by the three rows. We have labeled two separated regions along one track “DLi” (they are centered at different eccentricities). The borders of DLi were particularly sinuous in previous cases, making it possible to draw a straight line that intersects this area more than once (see e.g., Fig. 12).

The receptive field plots for row C (the most medial row) at the bottom of Fig. 17 show a series of seven reversals as the track crossed eight areas. The posteriormost penetration in each area is marked by the open arrows. Receptive fields start near the lower field vertical meridian inside V2 and progress to the horizontal meridian. They reverse upon entering DLp and return to near the lower field vertical meridian. There were no upper fields near the beginning of Row C, so we probably just missed recording from a small upper field DI and upper field DM (medial to penetrations 7–10) based on the position of the anteriorly-bending cytochrome oxidase-dense region we have tentatively identified as DM+. Receptive fields then return to the horizontal meridian, but at greatly reduced eccentricity. We labeled this region DLi on the basis of field sign and light cytochrome oxidase staining. Receptive fields reverse at the horizontal meridian and move back into the lower field, almost parallel to the vertical meridian in area “Z”. They then reverse again near the lower field vertical meridian, eventually reaching the horizontal meridian. We also labeled this region DLi (this sequence is at a greater eccentricity than the first DLi sequence). Beyond DLi in DLa/MTc, receptive fields once again return to the lower field vertical meridian. Receptive fields reverse at the lower field vertical meridian border of MT to return to the horizontal meridian, this time continuing into the upper field, where they eventually reach the upper field vertical meridian. Once there, receptive field size increases, and the sequence reverses for the seventh time, returning to the horizontal meridian and continuing slightly into the lower field in FSTd.

The receptive fields for the two successively more lateral tracks (rows B and A) are also shown at the bottom of Fig. 17. The sequence of reversals is similar, except that the deviation toward the center of gaze in area “Z” is less pronounced (we omit area “Z” and the second DLi in rows B and A). These more lateral rows resulted in receptive fields in each area that were at successively smaller eccentricities.

#### Field sign map and transparent overlay (Figs. 18 and 17 top)

**Fig. 18.**
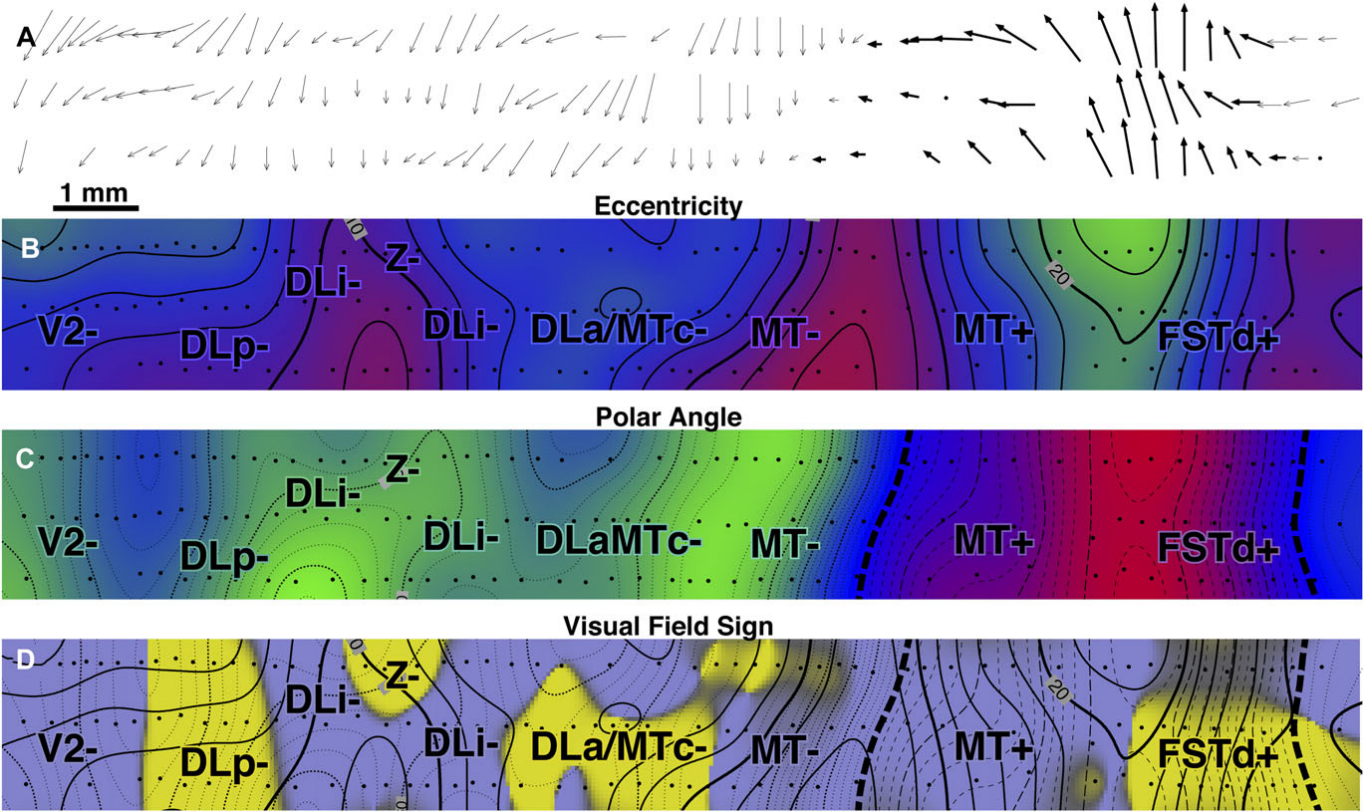
Arrow diagram, eccentricity, polar angle, and field sign maps for case 4 (V2 to FST). Four parallel representations of retinotopy are shown together for easy comparison (see Fig. 17 for corresponding recording locations and receptive field plots). The marked systematicity of retinotopy is clear in comparing the nearby arrows both within and between rows in (**A**). The isoeccentricity plot in (**B**) shows two minima—one in DLi and one near the DLa/MT border and one major maxima at the MT/FSTd border. The isopolar angle plot in (**C**) shows a lower field vertical meridian at the DLp/DLi border and the DLa/MT border, and an upper field vertical meridian at the MT/FSTd border. The visual field sign plot in (**D**) shows a regular alternation in field sign in crossing V2, DLp, DLi, DLa/MTc, MT, and FSTd. The two additional reversals in the first row result in an additional small finger of mirror image field sign protruding into DLi from the medial side at “Z”. The visual field representation in FSTd is quite distorted (isoeccentricity lines nearly parallel to isopolar angle lines), which contrasts with the more nearly orthogonal relation seen in the other areas.

In Fig. 18, the receptive field data from the three rows A–C is shown as an arrows diagram (A) rotated so that the rows are horizontal. The receptive field reversals just described are more easily viewed by following the angle of the arrows in each row. The penetration density in these three rows was locally the densest of any of the cases. The receptive field sequences are nevertheless very smooth, both within and between rows. Below the arrow diagram is the interpolated isoeccentricity map (B) (same scale as previous Figures: red central, blue middle, green high eccentricities). The range of eccentricities spanned by the recording area (8–22 deg) is less than in the previous illustrations so the color scale was expanded. Receptive fields approach the center of gaze in DLi and MT, and reach the periphery only in MT. The isopolar angle map is shown in C (green lower, blue middle, and red upper field). The posterior edge of the map is near the V1/V2 vertical meridian (green). Moving anteriorly, there are two lower field vertical meridians (green bands—the DLp/DLi border and the DLa/MTc to MT border), four horizontal meridians (blue bands—the V2/DLp border, the DLi to DLa/MTc border, and the horizontal meridian representations of MT and FSTd), and one upper field vertical meridian (red band—the MT/FSTd border). The vertical meridian band at the DLp/DLi border splits into “Y” as a result of the two extra reversals in the first row. The field sign map is shown at the bottom (D). It demarcates areal boundaries and illustrates the splitting of DLi into a Y-like patch with area “Z” between the arms of the Y. An inspection of the arrows diagram shows that receptive fields moved very rapidly toward the horizontal meridian at this point. On the basis of the previous cases, the upper field representation of DM or VPP lay just medial to “Z”.

In Fig. 17, top, the visual field sign map (yellow and blue–purple) from Fig. 18 has been transparently overlaid on the cytochrome pattern (gray shading). There was good agreement between the alternations in field sign and the cytochrome oxidase features previously mentioned; most field sign transitions occurred within 100–200 microns of the location of the areal border estimated from the cytochrome oxidase data. The pattern of field sign was complex near the MT border. This was similar to our previous cases and may reflect a complex pattern of retinotopy near the large cytochrome oxidase blobs in DLa/MTc.

A number of recording sites from near the center of gaze were also illustrated as a separate lateral patch of transparent field sign shading. This region contains receptive fields very near the center of gaze, which makes it much more difficult to resolve retinotopy, and especially gradient directions. V1 and V2 were nevertheless identified (the deviation from the architectonic borders likely reflects noise in the gradient estimates). Anterior to V2, receptive fields moved away from the center of gaze somewhat. There was evidence for two strips of alternating field sign, labeled VP and VA. These two strips had medial lower fields and upper field representations laterally.

It was difficult, however, given the unfortunate gap in the data, to be sure whether we might have missed some additional upper fields in lateral DLp and DLi like those seen in case 1 (Fig. 5, just below “DLp-” label and below “DLi+” label) that were disconnected from the large stretch of upper fields in ventral VP and VA. One intuitively attractive possibility is that the pairs DLp and VP, and DLi and VA each form a single area (see Discussion). This is supported by the observation that cytochrome oxidase was denser in VP than in VA (as it was in DLp *vs.* DLi). Another possibility is that DLp and DLi have small lateral (ventral) central upper field representations and VP and VA have small medial (dorsal) central lower field representations; thus, the medial to lateral retinotopic sequence might be: large lower field representation (DLp-, DLi-), small central upper (DLp+, DLi+), small central lower (VP-, VA-), large upper field representation (VP+, VA+). The receptive fields in the *medially* located DLp+ are all beyond 10 deg eccentricity.

### Case 5—dorsolateral cortex, cytochrome oxidase (52 sites)

Long mapping experiments such as the previous one tend to obscure the cytochrome oxidase pattern. Therefore, in this experiment, the recording session was terminated after only 10 h—preserving more detail in the cytochrome oxidase pattern, and reducing artifacts from erythrocytes. We were able to make a clear distinction here between the “thin” and “thick” stripes (both are nearly the same thickness, but alternate “thin” stripes are slightly darker). Despite limited coverage, we nevertheless uncovered evidence for a systematic alternation in field sign in the three DL areas between V2 and MT consistent with the previous cases.

#### Cytochrome oxidase pattern (Fig. 19, top)

As before, V2 bulged anteriorly near the end of the Sylvian, and the most lateral part of the presumed DM dense patch turned anteriorly. DLp was less densely stained than DM but more densely stained than DLi (though there were several small densely stained patches within DLi near penetrations 23 and 24; curiously, these also appeared in the case 4—near penetrations 127–130). DLa/MTc roughly corresponded to the region of large blobs surrounding MT, and MT showed evidence of medium sized blobs. TP appeared as a small, triangular, densely stained region along the medial border of MT, and there were several small stained regions along the lower lip of the Sylvian sulcus partly overlapping the expected location of TA and TD.

**Fig. 19.**
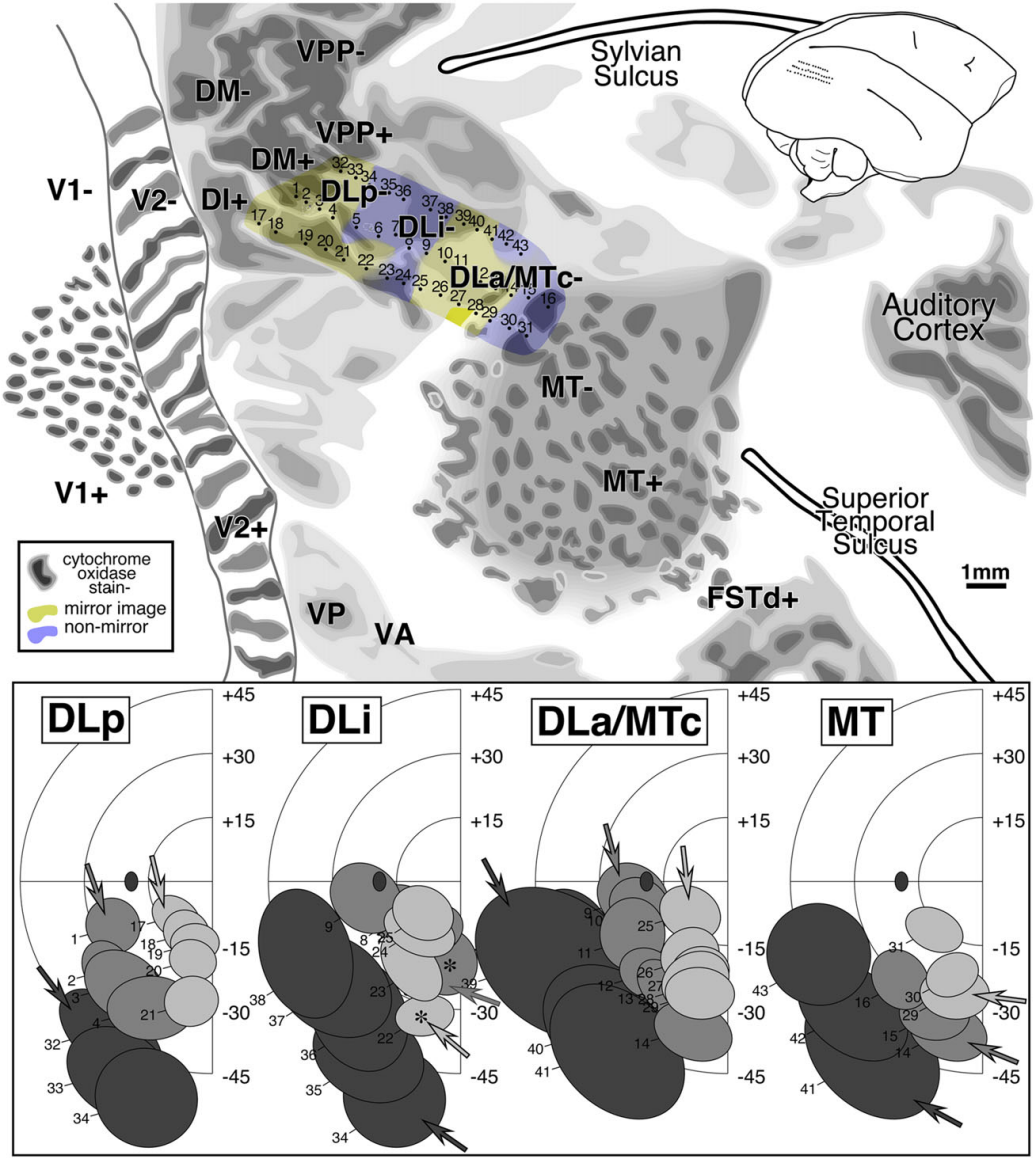
Case 5 cytochrome oxidase pattern, penetration sites, visual field sign (top) and corresponding receptive field plots (bottom). This experiment was terminated earlier to better preserve the cytochrome oxidase pattern at the expense of penetration density. Many features visible in the Fig. 17 were confirmed in this case. The V2 stripes were divisible into two sets (equal in width), one of which stained more darkly for cytochrome oxidase. A densely stained patch probably corresponding to DM adjoins V2 at the point where V2 suddenly thickens. The lateral parts of the putative DM patch turns anteriorly away from V2 moving laterally. DLp was more densely stained than DLi. DLa/MTc corresponded somewhat loosely with an irregular set of large blobs surrounding MT. TP was visible as a moderately dense cytochrome oxidase patch along the anteriormost part of the medial border of MT. There were several densely stained patches slightly more anterior and medial probably corresponding to TD and TA. The transparent visual field sign overlay shows 4 alternating patches of DLp (mirror), DLi (nonmirror), DLa/MTc (mirror), and MT (nonmirror) as expected. There were two small anomalies—a small mirror image (yellow) patch extending into DLi and a nonmirror image blob (blue–purple) outside of MT near the DLa/MT border (under “MTc-”). Receptive fields for the three rows are at the bottom, shaded to indicate row (light most lateral) with filled arrows indicating the most posterior penetration in each area. There are four re-representations of the lower quadrant—in DLp, DLi, DLa/MTc, and MT. There was a small anomaly in DLi where receptive field sequences from the lateral and middle rows briefly crossed near the vertical meridian reversal at the DLp/DLi border (asterisks), which causes the field sign to flip inside DLi at point 22.

#### Receptive fields and field sign/cytochrome overlay for DLp, DLi, DLa/MTc, and MT (Fig. 19)

Three rows across DLp, DLi, DLa/MTc, and into MT showed clear reversals and four almost complete representations of the lower visual field. As before, the posterior end of the row in each area is marked by a filled arrow and parallel rows are indicated by shading (most lateral is lightest). Our rows were slightly medial to those in case 4, but started further anteriorly, and so once again, we just missed recording from DI+, DLp+, and DM+. There were two small anomalies. The first occurred within DLi where the receptive fields from the lateralmost row crossed the middle row just after reversing at the DLp/DLi vertical meridian. By the time receptive fields reached the horizontal meridian in DLi, they had returned to their expected position as the least eccentric of the rows. The second small anomaly occurred at the DLa/MT border where the vertical meridian reversal appeared “too early”—at penetration 42. These two anomalies show up clearly in the transparent field sign/cytochrome overlay at the top of the Fig. 19. The first results in a small region of inverted field sign in DLi [small yellow lateral patch in DLi otherwise expected to be nonmirror image (blue–purple)]. The “early arrival” of the MT border shows up as a small nonmirror (blue–purple) excrescence on the medial border of MT. Given the undulatory nature of the borders between areas revealed in our more comprehensive experiments, these anomalies were not unexpected.

## Discussion

The owl monkey, *Aotus trivirgatus*, is the only nocturnal monkey. It appears to be secondarily nocturnal (e.g., it lacks a reflective tapetum), since the presumed common ancestor of monkeys was almost surely diurnal (Szalay & Delson, 1979; Fleagle, 1988; Finlay et al., 2008). Because of its habit, it has a reduced emphasis on the center of gaze in the retina (Silveira et al., 1993) as well as a larger proportion of M retinal ganglion cells, when compared with *Cebus* or *Macaca*. The ratio of the area of MT to V1 is similar to other monkeys, but V2 is slightly reduced (Pessoa et al., 1992). The reduced emphasis on the center of gaze is particularly salutary from the point of view of retinotopic mapping experiments because it increases the proportion of the cortex from which meaningful retinotopic mapping data can be conveniently collected. However, because of its habit, it has sometimes been insinuated that the study of this animal is less relevant to the goal of understanding the evolution and function of the human primate brain. By contrast, we think it is particularly important to study several different primate species—especially including ones like owl monkeys with derived conditions (nocturnality)—because the human primate shows so many striking derivations from the primitive condition that we would eventually like to understand in an evolutionary context (Sereno & Allman, 1991; Sereno, 1991; Sereno, 1998; Sereno & Tootell, 2005; Sereno, 2014). We start by asking “what is a cortical area?”, discuss our findings first with respect to other experiments on owl monkeys and then other monkeys, and finish with some methodological considerations.

### What is a cortical area?

It is important to remember that a cortical visual area is a human label for the adult form of a local part of the neocortex after normal development. Everybody agrees that useful kinds of information for defining a visual area include (1) retinotopic organization, (2) architectonic features (neuron morphology and arrangement, molecular markers), (3) connection patterns, (4) physiological properties, and (5) effects of lesions. But there is less agreement about what specific criteria are required for a region to be considered a “single visual area”. Some specific criteria that have been proposed include: (a) an area should contain a topological (neighbor-preserving) map of the entire hemifield *or* separate topological maps of visual quadrants that are attached near the center of gaze (split horizontal meridian), (b) small movements across the cortical map should not result in discontinuous jumps in the visual field, which implies that the topological map should be either mirror image or nonmirror image, (c) an area should have the same set of neighboring areas, (d) an area should always have the same set of connections, (e) architectonic features should be uniform throughout the area. These criteria work well in V1 (at least in the binocular part) in primates. But starting with V2 and MT, exceptions are needed. For example, different areas appear to touch V2 in different primates; there is a sudden drop in myelination within MT in the peripheral representation. Moving beyond V1, V2, and MT, the situation gets more unruly.

In primate extrastriate cortex, different researchers have relaxed different individual criteria. For example, the upper and lower field representations anterior to V2 have been argued to be two different areas despite the fact that they represent nonoverlapping and incomplete parts of the visual field (Burkhalter et al., 1986); the intercalated stripes within V2 re-represent nearby parts of the visual field; different parts of a single area may not need to form a single topologically connected region (V3: Felleman & Van Essen, 1991), or be characterized by a single visual field sign (DM: Rosa & Schmid, 1995); areas may not represent the whole hemifield represented in V1 (this applies to virtually all extrastriate areas!); some areas have secondary discontinuities beyond the horizontal meridian split (area 18 and 19: Tusa et al., 1979); the “horizontal meridian” split may not actually lie on the horizontal meridian (Gattass et al., 1988). For the current study, we have given substantial weight to visual field sign (e.g., in distinguishing DI and DM). But others might prefer to put more weight on connections or myeloarchitecture or functional properties, which may suggest a different parcellation. Moving forward, it will be more important to explicitly state how different criteria have been weighted, particularly when different criteria suggest different parcellations.

Looking across a wider range of species, it is clear that visual areas vary quite substantially. This even includes the visual areas touching V1. It has been suggested that extrastriate areas might have been formed by duplication (Allman & Kaas, 1974). But in the case of rats and mice, it appears that cortex next to V1 may have actually subdivided itself into multiple areas; the reason for looking at it this way is that the marsupial outgroups for placental mammals have a continuous V2, as do most other placental mammalian groups that have been examined (primates, carnivores, ungulates, tree shrews, and even squirrels, which are sciuromorph rodents). This suggests that having many areas touching V1 may be a derived, not a primitive condition—a specialization found in some rodent groups. Yet another possibility is that two areas may be combined to form one area. All three of these possibilities (duplication, subdivision, fusion) suggest that one area in one species can effectively correspond to several areas in another. Understanding how this works will require a deeper understanding of development (cf. Dyer et al., 2009, on retinal development). These kinds of changes also change neighbor relations. Finally, these kinds of interspecies variations may also occur among individuals of the same species. For example, some humans, including the first author, appear to have a V3A that touches a portion of V2 directly without an intervening V3, rather fittingly, like owl monkey DI.

Finally, it is implicitly assumed that if a visual area has been given the same name in two different animals that it should have roughly the same *function* in those two animals. But it is worth considering poignant examples from the evolution of other body parts such as case of the inner ear bones in mammals; two are homologs of bones that form the jaw articulation in reptiles and one is originally derived from a portion of what was originally a gill arch (stapes) (Romer & Parsons, 1977). This suggests that the “same” (in the sense of homologous) visual area may acquire a different function in evolution.

Substantial work remains to be done to fully characterize the retinotopic organization of visual cortex. As new more powerful mapping methods begin to be exploited (see below), it will be important to keep an open mind about area names, area evolutionary relations, area variability, and area functions.

### Extrastriate areas in owl monkeys

#### Organization of V2

V2 contains a nonmirror image representation of the visual field but three kinds of stripe-like compartments, some of which project to different areas (DeYoe & Van Essen, 1988; Livingstone & Hubel, 1988; Zeki & Shipp, 1989; Sincich & Horton, 2005). Each compartment might therefore be expected to have its own representation of the visual field. There was evidence that the visual field representation in V2 was substantially expanded in a direction roughly perpendicular to the stripes, but there was no evidence for regular visual field sign reversals lined up with the stripes; thus, multiple representations within different kinds of stripes may be achieved by receptive field scatter. The V2 stripes were found to lie along spirals when projected back into the visual field. The anterior–posterior width of V2 varied by a factor of two, with a characteristic anterior bulge near the location of DI. It was difficult to distinguish the anterior V2 border architectonically at most points along it.

#### Subdivision of original area DL—DLa/MTc

In their original study, Allman and Kaas (1974) envisioned DL as a V2-like area almost completely surrounding a V1-like MT. We have divided the posterior medial two-thirds of this original large area into three parts—DLp, DLi, and DLa/MTc (Sereno & Allman, 1991). The anteriormost portion of DL, here labeled DLa/MTc, clearly corresponds to the anterior wings of the original DL. It contains a narrow mirror image representation of much of the lower visual field and at least part of the upper visual field. DLa/MTc shares a vertical meridian border with MT and its shared border with DLi is approximately the horizontal meridian. The smaller, laterally located upper field representation of DLa/MTc wraps part of the way around MT, forming a vertical meridian border with FSTd that extends perpendicularly from the lateral convexity of the MT. Our data suggested that DLa/MTc peels away from MT at this point and continues laterally for a short distance.

A similar area is particularly apparent in the flatmounted cytochrome oxidase section in Tootell et al., 1985 (their Fig. 9, unnamed); it appears to peel away from MT laterally in several other of their flatmounted cases. Neurons in this area are quite responsive—easily confusable with MT in this respect—though they are not markedly direction selective. DLa/MTc neurons also have more sustained responses to stimuli than neurons in MT; the “DL” recordings in Petersen et al., 1988 came from lower field DLa/MTc in one of our chronic mapping animals. Kaas and Morel (1993) relabeled this same area MTc in the course of a connectional and architectonic study of FST.

Since this area clearly constitutes part of the “wings” of the original Allman and Kaas DL, and since V4t (see below) had not yet been defined at that time, we have here continued to call it a part of DL by priority.

#### Two more subdivisions of original area DL—DLi and DLp

We divided the remaining area between V2 and DLa/MTc into two more areas—DLp and DLi. These areas share a vertical meridian representation. This agrees nicely with the callosal connection studies of Newsome and Allman (1980) and Cusick et al. (1984) who both showed a prominent diagonal band of callosal label in owl monkeys situated in the middle of greater DL, at the expected location of the DLp/DLi vertical meridian border.

#### Organization of area DM

In several previous studies on owl monkeys (Allman & Kaas, 1975; Krubitzer & Kaas, 1993), DM was described as a rectangular heavily myelinated area bordering V2 rostrally and ending on the medial wall just beyond the dorsal convexity of the brain. The most extensive mapping case reported here confirmed the previously proposed organization of the medial, lower field portion of DM. However, we were surprised to find that the upper field cortex lateral to lower field DM directly touching the anterior border of V2 has the *opposite* visual field sign from lower field DM. We labeled this region DI and argue that the upper field representation of DM bends anteriorly away from the anterior V2 border. Upper field DM was considerably smaller than lower field DM. The density of myelination fell off in the peripheral parts of the medial lower field as well as in the upper field representation—but remaining at least as densely myelinated as the surrounding cortical areas (V2, DI, VPP).

**Fig. 20.**
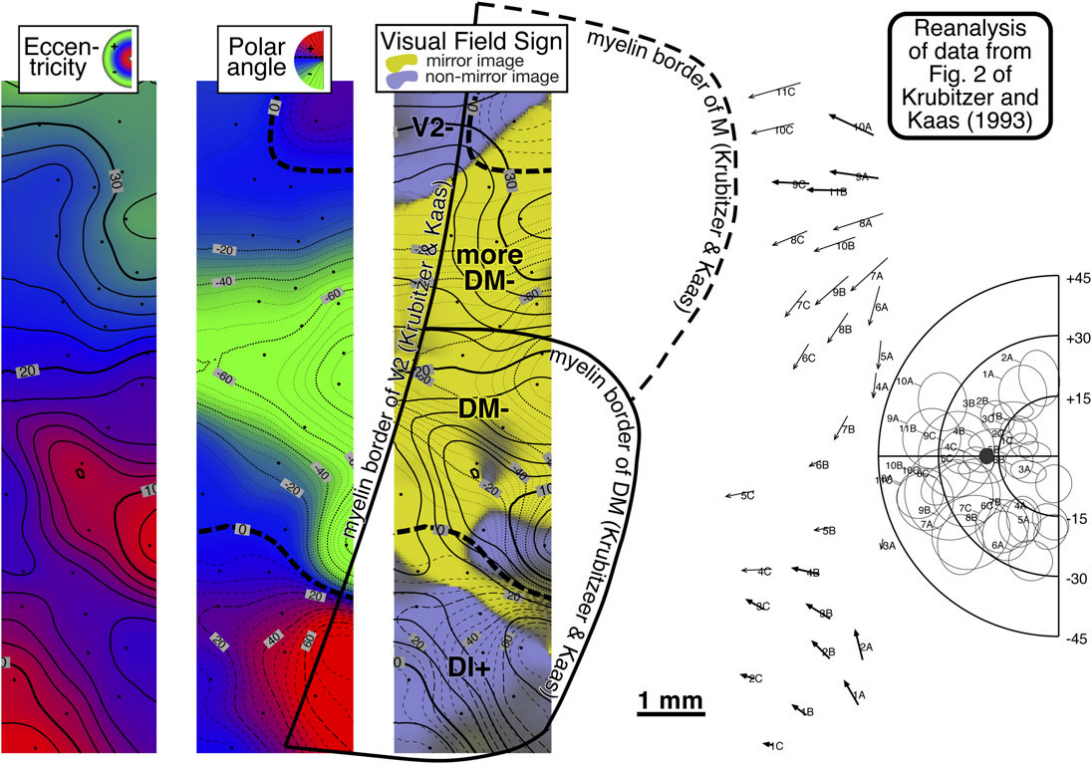
Re-analysis of DM/M data from Krubitzer and Kaas (1993). Data from Krubitzer and Kaas (1993, their Fig. 2) were digitized, interpolated, and illustrated in a similar manner to the data presented in this paper (receptive fields, arrow diagram, isoeccentricity, isopolar angle, field sign maps). Their original estimated borders are also shown. The field sign calculations show that visual field sign reverses within their architectonically-defined DM. As in our cases, visual field sign changed from mirror image to nonmirror image in the middle of the upper quadrant as one moved laterally (cf. the border between DM and DI in Figs. 12 and 16). The field sign map also shows mirror image cortex (lower visual field) continues into the area they labeled M. Our case 3 had a similarly elongated lower field representation in this region, all of which we labeled DM. We have applied new labels consistent with the present scheme. Nomenclature aside, it is clear their data was quite consistent with ours.

In Fig. 20, we digitized and reanalyzed the DM mapping data published by Krubitzer and Kaas (1993, their Fig. 2) using our visual field sign technique, and then superimposed their marked areal boundaries—the anterior borders of V2 and DM (solid contours) and the M border (dashed contour). Their retinotopic mapping data are consistent with the retinotopic organization of DM and DI proposed in this study (our re-labeling is indicated by DI+, DM-, “more DM-”, and V2-). Our re-labeled DM extends across their DM/M border as an elongated patch of mirror image lower visual field. Far laterally (bottom), there is a transition to nonmirror image cortex soon after receptive fields cross into the upper field within their myeloarchitectonically defined DM. This is similar retinotopically and myeloarchitectonically to what we saw at the DM/DI border in cases 2 and 3, suggesting that their upper field DM might mostly be DI as we have defined it.

#### Organization of area VPP

We identified a nonmirror image area adjoining DM anteriorly. Following Krubitzer and Kaas (1993), we labeled this area VPP; they showed it had strong connections with DM. As with DM, this area has an elongated medial lower field representation and a smaller, anteriorly bent upper field representation.

#### Organization of area DI

DI was originally defined as a third tier owl monkey visual area directly adjoining V2 situated between DM and DL (Allman & Kaas, 1975) with upper and lower visual fields, and a vertical meridian at its anterior border (Newsome & Allman, 1980). Krubitzer and Kaas' (1990; 1993) DI is centered at a similar location, but is shown as wrapping further around the anterior border of DM.

The present study identified area DI as a small area directly adjoining V2 that contains only the upper visual field and that has the same visual field sign as V2 (nonmirror image). In contrast with the original definition, Krubitzer and Kaas' version, and Rosa's marmoset DI (see below), we propose that DI actually lies *between* V2 and upper field DM. DI is relatively heavily myelinated, and appears almost continuous with the heavily myelinated lower field representation of DM. As with the original DI, the anterior border of our new DI is the (upper field) vertical meridian. It is smaller than previous DI's, and the lateral part of its anterior border tilts posteriorly—like the nearby borders of DLp, DLi, and DLa/MTc. It eventually thins to almost nothing as it reaches the 10 deg eccentricity contour in V2.

#### DM *versus* V3

After the mapping experiments in the present study were completed and analyzed (but not yet published), an old controversy was revived over the proper names and configuration of areas in the region of the cortex in owl monkey and other New World Monkeys centered on what we have called DI and DM. One idea is that the region immediately anterior to owl monkey lower field V2 contains a thin, continuous, lower visual field V3 similar to macaque monkey dorsal V3. In this model, no upper fields directly contact the anterior horizontal meridian border of lower visual field V2; instead, receptive fields are postulated to reverse into the lower field toward the lower field vertical meridian along the entire border of lower field V2 (Lyon & Kaas, 2002; Lyon et al., 2002) save for only for the far lower field periphery.

In fact, the extreme density of penetrations in lower field V2 in case 3 (experiment date: 1991) was an explicit attempt to find out whether or not owl monkeys had a small macaque-monkey-like “dorsal V3” that had somehow previously been missed. It is clear from case 3—but also case 2—that for 2–3 mm along the V2/DI border, there is no trace of a reversal back into the lower field immediately anterior to V2; receptive fields instead go into the upper field. Further medially, this reversal into the lower field *does* occur along at least 5–6 mm of the lower field V2/DM border part of which is on the medial wall; and it also occurs lateral to DI, until the center of gaze is reached.

It is unlikely that we simply misestimated the horizontal meridian given that receptive field centers progress to at least 20 deg of polar angle into the upper field (at an eccentricity of 10–20 deg) before reversing into the (small) DLp upper field. In anatomical experiments, this small upper field might have been differently interpreted because the anterior border of V2, which as we have shown is difficult to estimate by architectonics alone, was placed too far posteriorly; as can be seen in our cases 2, 3, 4, and 5, there is a considerable anterior bulge in V2 near the location of DI. Thus, the upper field connections revealed by Lyon and Kaas (2002) in the region of DI (e.g., their Fig. 8A and 8B), might actually directly abut V2. Although some optical recording retinotopy experiments have been done in owl monkeys (Lyon et al., 2002: horizontal meridian, vertical meridian, lower field only), an experiment that included upper field-only stimuli, or better, a thorough-going phase-encoded approach (Garrett et al., 2014; see below), would be required to disconfirm the picture presented here.

#### Organization of area TP

We identified a small mirror image area on the anterior part of the medial MT border that adjoins the anterior end of DLa/MTc. Architectonically, it was characterized as a small lightly myelinated but cytochrome oxidase-rich area. It has the same field sign as DLa/MTc; it was distinguished from DLa/MTc because it contains a re-representation of much of the lower visual field. Also, its upper field representation extends medially in contrast with DLa/MTc's lateral upper field.

#### Organization of area TA and TD

TD lies medial to TP while TA lies lateral and slightly anterior to TP. These two areas were found to correspond reasonably well with several densely myelinated patches along the lip of the Sylvian fissure that extend almost to the anterior border of MT. Both of these areas were identified as nonmirror image areas.

#### Organization of area MSTd and MSTv

MSTd was mapped in detail here, as well as in our chronic recording experiments (data not shown). It consisted of a small mirror image area sharing a representation of the periphery with nonmirror image MT. The center of gaze representation of this area lay almost directly anterior to MT (shared with TA). There were too few receptive fields from MSTv to make a definitive statement about retinotopy and visual field sign there.

#### Organization of area FSTd and FSTv

These small areas are situated anterolateral to MT. Mirror image FSTd, which lies just beyond the anterolateral border of MT, was systematically mapped in the present study. It contains a reasonably orderly, somewhat elongated representation of much of the visual field. We obtained a smaller number of receptive fields from more laterally located FSTv, most of them near the center of gaze, which suggested that FSTv is a nonmirror image area and contains a lower and upper field representation.

### Comparisons with other monkeys

The major similarities and differences with other monkeys, both New World and Old World, are summarized here. A comprehensive comparison with other primates including humans (e.g., Sereno et al., 1995, 2001, 2012; Huang & Sereno, 2013) is postponed to another study.

#### DL subdivisions resembling vertical-meridian-separated DLp (mirror image) and DLi (nonmirror image)

The region between MT and V2 has also been divided into two parts in a series of connectional studies in New World squirrel monkeys (Gould et al., 1987; Cusick and Kaas, 1988; Steele et al., 1991; Weller et al., 1991). Gould et al. (1987) demonstrated the existence of a prominent band of presumed vertical meridian callosal connections in the middle of the large original DL in squirrel monkeys similar to that seen in owl monkeys by Newsome and Allman (1980) and Cusick et al. (1984). Steele et al. (1991) showed that an injection on the DLc/DLr border labeled cells precisely on the shared vertical meridian representations of V1/V2 and DLa/MT (their Fig. 8). The laterally placed injection in that case appears quite close to V2, suggesting that the DLc/DLr vertical meridian representation is tilted in a similar fashion (lateral end tilted posteriorly) to the DLi/DLp border in owl monkeys revealed in the present study and the callosal band at the same location (Fig. 16; Gould et al., 1987, and Fig. 16 in; Cusick & Kaas, 1988). Intriguingly, the Steele et al. (1991) case (their Fig. 8) showed another patch of label adjoining MT laterally that may represent the vertical meridian border between lateral DLa/MTc and FSTd revealed in our mapping experiments. DLa/MTc was not explicitly identified in the studies of Weller et al. (1991) and Steele et al. (1991) and was likely included in their wider DLr.

A series of studies in marmosets (Rosa & Tweedale, 2000; Rosa et al., 2009; Jeffs et al., this volume), Rosa and Angelucci and colleagues identified two areas, VLP and VLA, whose lower field parts are very similar to owl monkey DLp and DLi (and squirrel monkey DLc and DLr). Medially, marmoset VLP contains only lower fields. The region we labeled DLp+ may correspond to marmoset DI+. A similar abrupt shift in receptive fields into the lower field is found moving just lateral to this region.

Connectional studies in squirrel monkeys (Cusick & Kaas, 1988, and; Steele et al., 1991) showed that the posterior subdivision, DLc (like mirror image owl monkey DLp) had strong input from V2, but the anterior subdivision, DLr (like nonmirror image owl monkey DLi) did not. We made similar observations in two retinotopically mapped owl monkeys (unpublished). However, Jeffs et al. (this volume) found that V2 projects equally strongly to both DLp-like VLP and DLi-like VLA in marmosets. One possible explanation for this discrepancy (aside from a species difference) is that the original distinction might have been due to lack of control over which V2 stripe type was injected.

It is not immediately obvious how to reconcile this New World monkey picture of two lower field DL areas, one of them strongly V2-recipient (mirror image), joined along the vertical meridian, with the standard picture of a single large V2-recipient V4d in Old World macaque monkeys with nonmirror image field sign that runs anteriorly all the way to meet V4t at a horizontal meridian. The possibility of subdivisions within V4 dates back to the original definition—the prelunate gyrus area of Zeki (1969) had two subdivisions, V4 and V4A, which were later combined into the “V4 complex”. However, there is no evidence inside “greater V4” of a prominent callosal band like the one separating DLp and DLi (or DLc and DLr) (Felleman et al., 1986). One possibility is that DLp is like V3d (both mirror image) and V4 is like DLi (both nonmirror image) (Rosa & Tweedale, 2000; Ungerleider et al., 2008). Note that this leaves V2-adjoining New World monkey DM without an obvious homolog in macaque monkeys. Another point is that in contrast to macaque V3d, owl monkey DLp bends anteriorly away from V2 as one moves medially in order to accommodate DI. Finally, owl monkey DLp appears to contain a small upper field that borders the upper field of DI at the vertical meridian; but no upper fields (DI or DLp+) are usually reported in this position in macaque monkeys (but see below).

Several recent fMRI retinotopy studies in macaque monkeys using surface-based phase encoded techniques and visual field sign analysis (Janssens et al., 2013; Kolster et al., 2014) present a different picture of macaque visual areas that in certain respects much more closely resembles our owl monkey data (N.B.: eccentricity and polar angle color schemes are reversed or rotated, and meridian line textures are different than here). Janssens et al. (2013, their Fig. 2) illustrate a pair of unnamed areas adjoining dorsal lower field V2 that look like lower field V3d (mirror image) and V3A (nonmirror image, also containing upper fields). At its medial end, the area they label V3 (mirror image) adjoins the anterior border of the two unnamed areas while more laterally, nearer the center of gaze, their V3 touches the near central lower field horizontal meridian of V2. In front of the dorsal area labeled V3 are two additional strip-like lower field areas labeled V4 (nonmirror image) and V4A (mirror image). The medial end of V4A contacts MT (nonmirror image). They illustrate a V4t that is actually lateral to MT and which contains upper fields. Except for the lack of upper fields directly touching V2, this configuration of areas looks much more similar to our Fig. 2 summary diagram (if area labels are ignored) than does the standard Felleman and Van Essen (1991) map.

#### DLa/MTc/V4t

There is clear evidence for an area like owl monkey DLa/MTc across many primate species. Rosa and Elston's (1998) detailed retinotopic map of the cortex immediately surrounding MT in marmosets revealed an MTc that is very similar to owl monkey DLa/MTc. They illustrate MTc as wrapping entirely around MT, but noted that the representation of polar angles in MTc was sometimes irregular, with local doubling back, particularly in portion of the lateral wing where we have illustrated DLa/MTc peeling away from MT (see the rows in two different animals that both start with receptive field 22 in the top and bottom of their Fig. 11). This area is extremely difficult to map given that its full width from the vertical meridian representation touching MT to its outer horizontal meridian border can be less than 1 mm across (Fig. 4A), which is less than the thickness of the cortex.

An area similar to owl monkey DLa/MTc was named V4t in macaque monkeys (Desimone & Ungerleider, 1986; Gattass et al., 1988) and DZ in cebus monkeys (Fiorani et al., 1989). These areas as initially defined only contained a lower visual field representation. V4t and DZ have the same retinotopic organization as lower field DLa/MTc (mirror image visual field representation, shared vertical meridian MT, posterior border is horizontal meridian), and stain somewhat less darkly for myelin and cytochrome oxidase than MT, but somewhat more densely than the surrounding cortex.

Several groups have provided retinotopic evidence for a human DLa/MTc/V4t similar to the nonhuman primate area, including Tootell et al. (this volume).

#### DI, DM, V3d, VVP, PIP

Though there was variation in this region between individual owl monkeys (e.g, DI in case 2 was roughly four times the area of DI in case 3), there were in both cases at least 2 mm of the anterior V2 border directly in contact with upper fields with no evidence of intervening progress back toward the lower field vertical meridian. A qualitatively very similar picture of the retinotopy in this region has been obtained in the New World marmoset monkey by Rosa and colleagues (Rosa and Schmid, 1995; Rosa and Tweedale, 2000, Rosa et al. 2005, 2009; Jeffs et al., this volume)—this despite the contrast in habit (marmosets are diurnal) and the fact that the marmoset retina looks a lot more like that of a macaque monkey than that of an owl monkey (Finlay et al., 2008; Dyer et al., 2009). A visual field sign analysis performed by digitizing the receptive field data from case CJ3 in Rosa and Schmid (1995) shows a patch of upper fields touching V2 that are nonmirror image, like our DI+. The main difference between our Fig. 2 summary diagram and theirs (Rosa & Schmid, 1995, their Fig. 17) reflects different criteria for deciding which regions of locally consistent visual field sign should be grouped together to form areas (see below). Rosa et al. suggest that despite the fact that their owl-monkey-DI-like DM+ has the opposite visual field sign from the elongated, medially-situated lower field DM, it may in fact be a displaced and flipped part of DM based on connectivity and architectonics (Rosa et al., 2005, their Fig. 18; Jeffs et al., this volume). By contrast, we have grouped a small anterior bending patch of mirror image upper fields (similar to part of marmoset mirror image DA+) together with V2-adjoining mirror image lower field DM (our VPP+ bends anteriorly as well); and we group DLp+, which resembles marmoset DI+, with DLp-. Retinotopy in this region is quite complex; we found four small, re-representations of the upper field within a few millimeters of each other (DI+, DLp+, DM+, and VPP+) here, and possibly an actual discontinuity. Given that it is difficult to combine multitracer connectivity studies with extensive high-density microelectrode mapping, using phase-encoded optical recording for retinotopy instead (see below) might be the way forward. In the interest of clarity, we have perhaps over-emphasized field sign; but we realize it is only one of several measures.

Finally, a study of MT connections in *Cebus* monkeys (Rosa et al., 1993) shows upper field projections from MT to a location extremely close to lower field V2 (e.g., their Fig. 10A). Their summary diagram shows an upper-field-containing V3A very close to V2.

Moving to Old World monkeys, the lower field part of our DM has a number of similarities with macaque area V3d (elongated representation, same visual field sign, dense myelination, similar input and output connections, similar bordering areas – see e.g., Felleman and Van Essen, 1987). However, there is little electrophysiological data unambiguously suggesting that any part of dorsal V3 in macaques contains upper visual fields that directly touch V2 the way owl monkey DI+ (or Rosa et al.'s marmoset DM+) does; instead upper fields in this region in macaque monkeys have only typically been seen further anterior in V3A, and only after a reversal back into the lower field (Gattas et al., 1988). However, it has been suggested that V3 is broken up into islands (Felleman & Van Essen, 1991; Kaas et al., this volume) so perhaps there is room for a small macaque DI in between the V3 islands. And see Angelucci and Rosa (this volume) for a reinterpretation of some of the recent macaque monkey fMRI data; a similar reinterpretation could be applied to Janssens et al., 2013 (described in “DL subdivisions” above).

In essence, the DM *versus* V3 debate is really about which cortical regions go together. One idea is that the (mostly) lower field region immediately anterior to dorsal V2 (V3d or DM) should be grouped with the mostly upper field area (V3v or VP) immediately anterior to ventral V2 (Zeki, 1969; Gattass et al., 1988; Lyon & Kaas, 2002; Kaas et al., this volume). Another idea is that dorsal V3 (or DM), and ventral VP are in fact different areas despite the fact that they contain non-overlapping parts of the visual field (Burkhalter & Van Essen, 1986; see Zeki, 2003, for a spirited riposte), and that the ventral upper field area VP in fact goes together with the posterior member of the pair of dorsal DL lower field areas (VLP in Rosa & Tweedale, 2000; Angelucci and Rosa, this volume), in a similar fashion to the pairing of macaque ventral V4 (or VA) with dorsal lower field V4d. The parcellation presented here is consistent with that second picture. In this way of thinking, the odd and apparently somewhat “unstable” region around DI, which contains one of the few examples we or others have found in the visual system of what could be an actual discontinuity—i.e., where nearby points in the cortex have receptive fields that do not overlap—might reflect a region in which different species—or even different individuals within a species—have settled on slightly different arrangements.

Moving anterior to DM, owl monkey VPP is similar to an area anterior to dorsal V3 that has been given several different names including PIP (Felleman et al., 1987; Colby et al., 1988) and DA (Rosa & Schmid, 1995). Finally, anterior to VPP is PP (which resembles marmoset PPd); it may be similar to macaque area LIP on the basis of connections, myelination, and location near but not directly abutting the intraparietal crease.

#### Small areas surrounding the anterior end of MT

The two subdivisions of FST and MST have here been given names originally applied to similar macaque areas because of the similarity in position and connections (Boussaoud et al., 1990; Kaas & Morel, 1993).

TP as defined in the present study, however, has no obvious macaque equivalent. Geographically, it would be expected to lie between the dorsal end of V4t and MST in macaques. Cortex in this region received projections from MT in both owl monkeys and macaques (Weller et al., 1984; Ungerleider & Desimone, 1986).

TA and TD could be roughly comparable to the dorsal prelunate area (DPL), area 7a, and perhaps, the dorsalmost part of MST. In marmosets, TA and TD correspond to small anterior parts of a large PPv.

### Position of the vertical and horizontal meridian

Visual cortical maps have often been defined as regions bounded by the vertical or horizontal meridian. The terms vertical and horizontal meridian, however, have been used to refer to different things in the visual field, retina, and cortex.

#### Vertical meridian

The retinal vertical meridian decussation is well-defined for a given cell class in the retina. However, different cell classes behave differently; and visualizing the retinal decussation in a mapping study is difficult. But more critically, the retinal vertical meridians by themselves do not determine how the animal holds these meridians with respect to each other in the visual field. To electrophysiologically define the visual field position of a particular cortical border (e.g., the V1/V2 “vertical meridian” reversal), the conscious animal would have to hold both eyes in their normal position during fixation while receptive field sequence reversals were plotted at the V1/V2 border in both hemispheres, through each eye separately—i.e., four reversals. None of those four are guaranteed to coincide; and there is evidence in several animals that they can differ (e.g., the peripheral lower field V1/V2 reversal is 20 deg into the ipsilateral field in ground squirrels—Paolini & Sereno, 1998).

With an anesthetized animal, two of the four reversals remain well defined—the cortical vertical meridian (e.g., the V1/V2 border) in both *hemispheres* through one *eye* (Hughes & Vaney, 1982; Payne, 1990; Sereno et al., 1991). This makes it possible to determine the shape (though not the absolute position) of the *monocular* cortical vertical meridian representation, and to determine what portions (if any) of the visual field are represented bilaterally *for that eye*. But this technique cannot determine the relative visual field placement of left and right hemisphere cortical vertical meridian reversals recorded through the *left* eye and those recorded through the *right* eye since information about how the animal holds its eyes in relation to each other is missing.

One could record from matching parts of the geniculate and then assume that left and right eye receptive fields coincide. However, sampling exactly corresponding points in the two laminae perpendicular to the local tangent plane is very difficult.

Finally, in areas beyond V1, receptive fields increase in size. Since maps are typically based on receptive field centers, vertical and horizontal meridian reversals occur at a greater distances from the actual meridians. Massed plots of receptive fields suggest that larger receptive fields tend to extend somewhat *less* than half their diameters beyond the vertical meridian border defined by smaller receptive fields. Thus, even monocular vertical meridians differ in different areas.

#### Horizontal meridian

The horizontal meridian is even more difficult to define. Unlike the vertical meridian, it has no direct retinal expression in an animal with an approximately radially symmetric distribution of retinal cell types. It comes into existence only in the cortex *via* the bifurcation of connections from V1 to V2 and from V1 to the parts of the pulvinar. One operational definition is the line orthogonal to the vertical meridian that passes through the peak in retinal ganglion cell density and/or the optic disk. But it is not clear why V1 to V2 connections must bifurcate along a straight line in the visual field, be orthogonal to the vertical meridian (itself not a straight line), pass through the representation of the optic disk, or even be positioned the same from animal to animal. And several studies have suggested that the anterior border of V2 is variable and does not lie directly on that operationally defined horizontal meridian (Gattass et al., 1988; Rosa et al., 1994).

#### Local visual field sign is unaffected by position of the meridians

A substantial advantage of a relative measure like local visual field sign is that it does not rely on knowing “actual” receptive field coordinates (i.e., defined binocularly in the awake fixating animal). All that is required is that the *relative* positions of receptive fields recorded through one eye be accurately known. Local visual field sign is invariant to rigid transformations of the receptive field coordinate system because these transformations do not affect the gradients in eccentricity and polar angle that are used to calculate visual field sign (see Sereno et al., 1994).

### Sources and effect of noise in retinotopic mapping data

Assessing the validity of our results requires estimating measurement errors in cortical *x*–*y* and visual field *r*-*θ* from their actual values and their effects on visual field sign calculations.

#### Noise in measurement of cortical *x*–*y*

The first source of *x*–*y* noise arises during marking penetration locations on the photograph. Since the smallest pial surface vessels are separated by 100–200 microns, these errors were probably restricted to a 50 micron radius of the true location.

A second source of position error comes from the viewing axis of the cortex photograph (which was taken along the electrode axis) not being everywhere locally perpendicular to the cortex, which is unavoidable because the cortex is not completely planar. These errors are a function of the *cosine* of the angle between the camera's line of site and the surface normal, and so are small; and they are spatially smooth. The offset from local surface perpendicular was under 4 deg (negligible position error) for most penetrations, only rising above 8 deg for a few penetrations at the craniotomy edge and and at sulcal lips.

A third source of *x*–*y* errors arises from having to stretch the cortical photograph penetration locations to line them up with the final location of the lesions observed in the physically flattened tissue. Avoiding this by making a lesion at every recording site (cf. Gould et al, 1986) was not practical given our high penetration density. Instead, we placed 8–12 lesions per case and then applied a local-geometry-preserving stretching algorithm. The great majority of penetrations were within 3 mm of a controlling marker. We estimated these errors to have a similar magnitude to initial recording of position on the photograph.

Finally, because recordings were made at a cortical depth of approximately 700 microns, the recording site location can differ from the pial surface electrode entry point. Since these *x*–*y* errors are proportional to *sine* of the angle between the electrode and the surface normal, they are more problematic than the errors in locating the pial entry point on the photograph—e.g., 4 deg offset → 50 micron error; 8 deg offset → 100 micron error; 20 deg offset → 250 micron error. These errors are smooth; but they can cause problems in relating surface-location-derived visual field sign maps to depth-lesion-derived locations in myelin stained sections from deeper cortical laminae.

#### Noise in measurement of receptive field *r*-*θ*

Quantitative estimates of receptive fields show they are typically elliptical, but do not have sharp boundaries (see e.g., Albright & Desimone, 1987; Jones & Palmer, 1987). We plotted a “boundary crossing” when a cell cluster's response on the audio monitor rose above a certain threshold. The accuracy relies on the experimenter's audio–visuo motor performance as well as the level of excitability of the cortex—both of which can vary across a long experiment. These errors were minimized by sampling many border points (a minimum of 10–15 locations per receptive field) but then only considering the center of the receptive field ellipse for analytical purposes.

A second source of *r*-*θ* errors is eye movement. The anesthetized animals in these acute experiments were not paralyzed. Their large eyes were immobilized instead by attaching a form-fitting ring to the corneal margin with cyanoacrylate tissue cement, taking advantage of the poor mechanical advantage (relative to the ring) of the posteriorly inserting eye muscles of this nocturnal animal, as well as the muscle-relaxing effects of triflupromazine. The projected position of retinal landmarks, tested after every 10–30 receptive fields, remained stationary to within our measuring abilities (∼1 deg error) suggesting no systematic drift occurred.

A third source of *r*-*θ* errors arises during digitization. Each new set of 25–50 receptive fields was traced onto a spherical paper sheet so the hemisphere could be periodically cleared. To digitize receptive fields, sheets were positioned on a polar coordinate system using the multiple retinal landmarks. We estimated our accuracy here to be ∼1 deg. As an example of cross-sheet stability, all receptive field sequences shown in Fig. 9 are from rows sampled perpendicular to the original penetration rows; thus each successive receptive field came from a different sheet separated by hours or even days. The systematicity of that data resembles data from proper rows (e.g., Fig. 5).

A fourth kind of *r*-*θ* error arises from changes in the optics of the eye over time. The margin of the gas permeable contact lens was kept moist and locally anesthetized but otherwise undisturbed. Any gross changes in optics capable of substantially altering receptive field *r*-*θ* would have been expressed as changes in the position and distance between the replotted retinal landmarks; their stability suggested that these errors were also within the inherent accuracy of our reverse projection technique.

A final source of *r*-*θ* “errors” is temporary or permanent changes in the position of the receptive field due to short-term cortical or subcortical plasticity. In several cases, we replotted the position of a receptive field after re-penetrating a particular cortical point one or more days later. This was typically done blind (the receptive field plotter did not know which receptive field they were replotting). The second receptive field center was always very close to the first (i.e., within 1–2°). Note that this kind of test across different data sheets includes all types of *r*-*θ* errors as well as several of the *x*–*y* errors discussed previously. Using ketamine as an anesthetic, which blocks NMDA channels, may have helpfully limited cortical plasticity.

All the five kinds of errors described above should be distinguished from “real” noise in retinotopy, often called receptive field “scatter”, defined loosely as the degree of deviation of individual receptive field *r*-*θ* values from smooth, best-fitting *r*-*θ* surfaces.

#### Effects of *x*, *y*, *r*, *θ* noise on visual field sign

Visual field sign, *λ*, is a convenient local measure that can be computed for each small patch of cortex. However, because it is based on local differences (gradients of *r* and *θ*), it is susceptible to noise. Another difficulty is that microelectrode data cannot easily be taken on a regular grid. To overcome this, we used a distance-weighted averaging technique to smooth the data and interpolate it to a regular grid. This is equivalent to a low-pass spatial frequency filter. The half-height radius of the distance-weighting function was typically 600 microns, which was comparable to our penetration sampling distance.

Most of the noise sources in *x*–*y* measurements are smooth and would therefore have minimal deleterious effects on the overall pattern of field sign. For example, when we compared the field sign pattern calculated from unstretched data to that derived from the stretched-to-flatmount data, the local shape of boundaries was preserved despite the global deformation of the sheet. The main worry about *x*–*y* errors is instead that they can affect the accuracy of field sign/architectonic overlays; they are the most likely explanation for the small mismatches we observed between field sign and architectonic borders (see e.g., the MT/DLa border in Fig. 12 and the V1/V2 border in Fig. 16).

The amplitude of the *r*-*θ* errors is approximately constant across the visual field. However, since the amplitude of the *gradients* in *r* and *θ* are reduced near the center of gaze, we would expect our estimates of those gradients—and hence visual field sign—to be noisiest there.

Potentially the most troublesome *r*-*θ* errors for visual field sign calculations are systematic errors from combining data across different sheets of receptive fields; these affect groups of receptive fields (here, posterior to anterior rows of penetrations). That kind of error would produce artifacts in the field sign picture aligned with the recording rows. Our field sign maps seemed free of them.

### Rationale for alternation in field sign

The field sign technique relies on the fact that many adjoining visual areas (e.g., V1/V2) have opposite field sign. One reason might have to do with constraints on visual areal borders, which are often (perhaps always) congruent in the sense that receptive fields near the border but on opposite sides of it tend to be close to each other. Congruent borders on a 2-D surface require that adjoining not-too-distorted areas have opposite field sign. Congruent borders naturally arise in development because neurons on either side of a congruent border tend to be co-activated, driving a correlation-based synaptic plasticity rule.

Another reason for the ubiquity of alternating field sign might have to do with the geometry of interconnecting cortical areas from only one side of the cortical surface. If a bundle of fibers projecting downward into the white matter maintain their neighbor relations as they curve back up into an adjoining area, the resulting visual field representation will change field sign from the first area to the second. Now certainly, there is ample evidence that fibers in the white matter can resort themselves in order to retinotopically interconnect areas with the same visual field sign (see e.g., the V2 → MT projection, which connects a nonmirror area to another nonmirror area). Nevertheless, many “canonical” cortico–cortical connections are mirror-reversing (e.g., V1 → V2, V1 → MT, V2 → V4).

This may reflect a more general pattern. For example, Kaas and Morel (1993) demonstrated strong connections between a set of nearby areas with opposite field sign: FSTd (mirror) ↔ MT, V2, VPP (nonmirror) and FSTv (nonmirror) ↔ DLa/MTc, TP, DLp (mirror). This pattern was not absolute [see e.g., FSTd (mirror) ↔ DM (mirror)]. A more extensive survey could describe if this pattern predominates.

### Determining visual field sign from optical recordings

The experiments reported in this study (several with more than 500 recording sites) constitute some of the most exhaustive extrastriate retinotopic mapping experiments conducted to date in single animals. Nevertheless, ambiguities remain that can only be settled by sampling a substantially larger number of sites in a single animal. In an owl monkey, for example, it is possible to expose more than 400 mm^2^ of visual cortex (e.g., case 3). In our extensive chronic mapping experiments (data not shown), we sometimes found systematic changes in receptive fields while making 100 micron steps tangential to the cortical surface. To accurately resolve the borders of visual areas using retinotopy would therefore require one penetration every 100 microns of cortex in the x and y directions, or about 40,000 penetrations total (cf. density required to map fractured somatotopy in the cerebellum: Shambes et al., 1978). This is two orders of magnitude more sites than we were able to obtain—a total still out of reach of current single microelectrode techniques.

The visual field sign method (Sereno et al., 1994) was originally developed for analyzing microelectrode recordings, but then quickly adapted to mapping retinotopy in humans with fMRI by using phase-encoded stimuli such as expanding rings and rotating wedges (Sereno et al., 1995). More recently, these methods have been used to map retinotopy in mouse V1 using optical recording (Kalatsky & Stryker, 2003) and to calculate visual field sign in order to parcellate extrastriate cortex (Garrett et al., 2014). Those studies used linear stimuli better suited to the mouse visual system, which lacks a strong central emphasis. This method—but using rings and wedges, which are better adapted to the primate visual system—would be able to provide the 40,000 data points that would be needed for a definitive visual field sign map of lateral extrastriate cortex, with histological correlates, in a flat-brained monkey.

## Appendix

### Cortical area abbreviations

DI: Dorsointermediate area
DLa/MTc: Dorsolateral anterior area (MT crescent)
DLi: Dorsolateral intermediate area (squirrel monkey DLr, marmoset VLA-)
DLp: Dorsolateral posterior area (squirrel monkey DLc, marmoset VLP-)
DM: Dorsomedial area
FSTd: Fundal superior temporal sulcus area, dorsal division
FSTv: Fundal superior temporal sulcus area, ventral division
ITcd: Caudal inferotemporal area, dorsal division
ITcv: Caudal inferotemporal area, ventral division
ITi: Intermediate inferotemporal area
ITpol: Polar inferotemporal area
ITr: Rostral inferotemporal area
M: Medial area
MSTd: Medial superior temporal area, dorsal division
MSTv: Medial superior temporal area, ventral division
MT: Middle temporal area
PP: Posterior parietal area
TA: Temporoanterior area
TD: Temporodorsal area
TP: Temporoposterior area
VA: Ventroanterior area
VP: Ventroposterior area
VPP: Ventral posterior parietal area
V1: Striate cortex
V2: Second visual area
V3: Third visual area
V4: Fourth visual area
V4t: V4 transitional
Z: (uncertain)
Z1/Z2/Z3: (possible center of gaze areas)

### Other abbreviations

A-I: Primary auditory cortex
M-I: Motor cortex
R: Rostral auditory area
STS: Superior temporal sulcus
1: Somatosensory area 1
3a: Somatosensory area 3a
3b: Somatosensory area 3b
